# How credible are Okun coefficients? The gap version of Okun’s law for G7 economies

**DOI:** 10.1007/s10644-022-09438-9

**Published:** 2023-01-09

**Authors:** Martin Boďa, Mariana Považanová

**Affiliations:** 1 Faculty of Economics, Matej Bel University in Banská Bystrica, Tajovského 10, 975 90 Banská Bystrica, Slovakia; 2 Faculty of Natural Sciences, Jan Evangelista Purkyně University in Ústí nad Labem, Pasteurova 15, 400 96 Ústí nad Labem, Czechia

**Keywords:** Okun’s law, Gap version, Asymmetry, Hodrick–Prescott filter, Hamilton filter, Unobserved component model, (T)ARDL model, E24, E32

## Abstract

The paper draws attention to the fact that findings that follow from estimation of Okun’s law are extremely sensitive to methodological choices. The argument rests in a case study oriented upon G7 countries for a period 1991/Q1–2021/Q4 and accounts for a possible asymmetry in the output–unemployment relationship. First, business and unemployment fluctuations are estimated by six purely statistical approaches that arise by casting the Hodrick–Prescott filter, the Hamilton filter and the unobserved component model into a univariate or bivariate framework. Second, the gap version of Okun’s law is modelled by means of an auto-regressive distributed lag model or its nonlinear threshold counterpart according as asymmetry is allowed or not. The results indicate huge heterogeneity in Okun coefficients for every country caused by differences even in the basal methodological aspects accounted for in the case study. The diversity of results demonstrates that initial modelling choices may provide economic policy-makers with conflicting insights and advice. This issue follows merely from the absence of general standards that might decide which particular result is more credible.

## Introduction

In spite of lacking an accepted theoretical derivation, Okun’s law is generally considered to be a useful forecasting tool that relates fluctuations in unemployment to fluctuations in output (Mitchell and Pearce 2010; Ball et al. 2015; Pierdzioch et al. 2011). For this reason, it is presented in leading economic textbooks as one of the core principles of macroeconomics (Blanchard 2017, p. 54, but also Blinder 1997, p. 241) or appears in economic models as an element connecting aggregate supply with the Phillips curve (e.g. Snowdon and Vane 2005; Blanchard 2017, pp. 198–199). Okun’s law predicts in the manner of a simple regression model that an upturn in output happens alongside a downturn in unemployment, but avoids claiming the existence of any causal mechanism. Since its formulation by Okun (1962), Okun’s law has been found to hold, if with varying strength, for a number of countries. It is especially popular to estimate Okun’s law for a panel of countries in a comparative fashion using different methodologies (e.g. Moosa 1997; Freeman 2001; Zanin and Marra 2011; Ball et al. 2017; Ball et al. 2019; Boďa and Považanová 2021). Nonetheless, although the philosophy of Okun’s law is simple, its empirical implementation is hampered by a variety of choices that precede its estimation.

To begin with, there are two basic formulations that differ as to how fluctuations are handled: either as consecutive (annual or quarterly) changes in output and unemployment (i.e. the difference version), or as deviations from potential output or the natural rate of unemployment (i.e. the gap version). Whereas the former is straightforward, the latter requires that a method is adopted for the estimation of both the output and unemployment gaps (which is partially associated with the ambiguity what exactly is understood by potential output or the natural rate of unemployment). Essentially, two options are available. One option, suited especially to estimation of the output gap, is to specify a suitable economic model mimicking the production function in order to identify business and unemployment fluctuations (e.g. Parigi and Siviero 2001; Proietti et al. 2007; Proietti et al. 2020) or to postulate a structural model combining different aspects of economic policy (e.g. Vetlov et al. 2011; Kiley 2013). The other option is atheoretical and based on some empirical trend extraction method with a minimum input by economic theory (e.g. Ladiray et al. 2003), which makes it a prevalent approach. Nonetheless, trend extraction is troubled by a broad range of possibilities to extract the trend component and construct the gap variable and by an impossibility to provide insights suitable for policy-making (Congressional Budget Office 2004, pp. 5–6). Examples of widely used univariate methods include the filtering approaches developed by Beveridge and Nelson (1981), Hodrick and Prescott (1997) or Hamilton (2018) as well as the unobserved component models of Harvey (1989) or Clark (1987). These take real output and unemployment rate series and apply an identical methodology to them in isolation so as to capture cyclical oscillations, or the transitory component. Save for the Hamilton filter, there have been various multivariate extensions to these methods, such as the multivariate Beveridge–Nelson filter (Evans and Reichlin 1994), the multivariate Hodrick–Prescott filter with a multitude of forms (Laxton and Tetlow 1992; Dermoune et al. 2009; Poloni and Sbrana 2017) or a system-wise formulation of the unobserved component model (e.g. Apel and Jansson 1999; Kara et al. 2007). Some of these multivariate extensions take a semi-structural perspective, for instance, we directly implement macroeconomic regularities such as the Phillips curve or Okun’s law, often with a priori pre-set coefficients (e.g. Laxton and Tetlow 1992; Conway and Hunt 1997; Apel and Jansson 1999). Having obtained reasonable estimates of the output and unemployment gaps, the equation of Okun’s law can be estimated in a naïve fashion as a simple linear regression model (e.g. Ball et al. 2019) or perhaps with auto-regressive distributed lag effects (e.g. Ball et al. 2017). Sometimes, an explicit consideration is given to modelling structural breaks (e.g. Huang and Chang 2005), time-varying coefficients (e.g. Huang and Lin 2008; Kim et al. 2020), or asymmetries over the business cycle (Silvapulle et al. 2004; Cevik et al. 2013; Boďa et al. 2015). In other cases, estimation of the gap variables is carried out simultaneously with estimating Okun’s law in a state space time-varying framework (e.g. Clark 1989; Guisinger et al. 2018).

In the light of a variety of modelling choices that predate actual estimation of Okun coefficients and with a focus upon the methodologically more complex and general gap version of Okun’s law, this paper studies to what extent Okun coefficients that arise from different (statistical) approaches to estimating the underlying gap variables are comparable and can be trusted. The design is kept simple by confinement to three univariate and correspondent bivariate filtering methods to the identification of gap variables in combination with a regression specification with and without possible asymmetric effects. The motivation for this set-up comes from different considerations:

- First, it is no secret that estimated output gaps vary immensely with the chosen method (Ladiray et al. 2003; Chagny et al. 2004). It is partially owing to the fact that potential output is unstable (Congressional Budget Office 2004, p. 2) and a non-negligible say in the estimation is the very notion of potential output as an economic category. Kiley (2013, p. 1) summarizes three chief definitions of potential output, and these by their nature require different methodologies (see also Congressional Budget Office 2004, p. 1ff).
- Second, it seems that applied academic work is inclined more to simpler atheoretical statistical approaches with a limited role of background economic theory, whereas research at economic institutions prefers statistical procedures grounded in economic theory. The traditional choice is in favour of statistical filters, which are the subject of inquiry in this paper.
- Third, the filter of Hodrick and Prescott (1997) is undoubtedly applied most frequently amongst statistical filters regardless of the scathing criticism accumulated throughout the years (e.g. Harvey and Jaeger 1993; Cogley and Nason 1995; Hamilton 2018). A recent remedy is the filter of Hamilton (2018) that seeks to resolve spurious identification of cycles and applicational drawbacks. Yet, the usefulness of these two approaches is discussed, and the debate will scarcely near its end as is revealed in the recent explorations by Phillips and Shi (2019), Hodrick (2020) and Franke and Kukačka (2020). In addition, the unobserved component model in the manner of Harvey (1989) or Clark (1987) is almost as popular as the Hodrick–Prescott alternative in terms of popularity. These three univariate filtering methods are applied in the paper for comparative purposes and are addressed here as the HP, H and UCM filters, respectively.
- Fourth, it is generally known that additional information improves reliability of estimates of gap variables (St-Armant and van Norden 1997, p. 35; Ladiray et al. 2003, p. 51; Chagny et al. 2004) and that output gaps are closely related to unemployment fluctuations (Kiley 2013, p. 2; Congressional Budget Office 2004, pp. 2–3). This motivates simultaneous filtration for the output and unemployment gap and gives rise to bivariate extensions applied to output and unemployment series. The HP filter is considered in the form of Dermoune et al. (2009), the H filter is extended in a natural bivariate way, and so is the UCM filter like in a different context by de Winter et al. (2017) and Fadiga and Wang (2009).
- Fifth, asymmetric responsiveness of unemployment to business fluctuations in Okun’s law is another safely established fact that serves in explaining the time variance of Okun coefficients or the nonlinearity of the Okun equation (Silvapulle et al. 2004; Huang and Lin 2006; Marinkov and Geldenhuys 2007). Despite the availability of numerous methods to isolate the asymmetry in an Okun’s law relationship, the paper employs a threshold auto-regressive distributed lag (TARDL) model (used, e.g., by Boďa et al. 2015; Tang and Bethencourt 2017). TARDL regression is a simple and fully descriptive aid in assessing the extent of asymmetric effects that packages information on the asymmetry in a manner suited to economic policy. Perman et al. (2015, p. 106) designate threshold regression as the most frequent approach to modelling nonlinearities.

Bearing this in mind, the paper emphasizes that it matters what configuration of analytical choices is made at the onset of an Okunian analysis. The paper is shaped as a comparative case study with the aim of assessing comparability of Okun coefficients under the gap version arising from different methods of isolating the output and unemployment gap (three univariate and three bivariate filters) whilst accounting for asymmetries in sensitivity of unemployment to the phase of the business cycle. To this end, quarterly data for the period between 1991/Q1 to 2021/Q4 are used for the seven G7 countries. The utilization of standard and threshold ARDL models permits a satisfactory amount of comparability of the present results and Okun coefficients with many other studies whose analytical framework is grounded upon a linear framework, albeit not necessarily dynamic. Estimated Okun coefficients display high diversity, even though they are not at odds with the values reported by past studies. In addition, some filtering approaches point to the existence of asymmetries in Okun’s law, whereas some indicate that there is no such nonlinearity. There appears no uniformity or pattern behind the approaches.

It must be critically admitted that model uncertainty is a well-known issue in empirical modelling, but this topic in the business cycle literature has not been sufficiently appreciated in connection with Okun’s law. Whereas research on decomposition methods and their weaknesses, typically in relation to estimating output gaps, is extensive (e.g. Cogley and Nason 1995; Perron and Wada 2009; Kiley 2013; Grant and Chan 2017; Furlanetto et al. 2020), little is known how particular choices made at the initiation of an Okunian analysis affect the results. In addition to parameter uncertainty that can be evaluated through statistical significance, estimated Okun coefficients are also exposed to model uncertainty, which is explored here in a context of selecting a particular statistical approach to output and unemployment gap estimation. The position of the paper in the extant literature in this regard is unique. Several approaches to gap estimation in empirical research are occasionally applied with the intention of a robustness check, which was first considered by Lee (2000) and became later fairly customary (e.g. Ball et al. 2017). This cautious approach is not only the domain of studies on Okun's law, but is also common in studies of employment growth (e.g. Elroukh et al. 2020). For this purpose, in some cases various approaches to estimation of Okun coefficients are employed with a particular gap extraction method (e.g. Moosa 1997; Zanin 2021). A thorough study of sensitivity of estimated Okun coefficients to the choice of a gap estimation method has not been conducted yet. An exception is perhaps Arčabić and Olson (2019) who juxtaposed estimates of static Okun coefficients for 20 OECD countries derived from gaps estimated with the aid of the Hamilton and Hodrick–Prescott filter in order to discover that contemporaneous Okun coefficients estimated from gap variables yielded by the Hamilton filter are greater in magnitude.

After this introduction containing a basic literature survey, the remainder of the paper consists of four more sections. Whilst Sect. 2 explains the filtering and modelling framework, Sect. 3 describes the data and presents results. Finally, Sect. 4 discusses and Sect. 5 concludes.

## Empirical strategy: filtering and modelling techniques

Okun’s law posits that output and unemployment fluctuations are negatively correlated and describable by an equation whose basal linear representation can take form:

1 $$ u_{t}^{c} = \alpha + \beta y_{t}^{c} + \varepsilon_{t}^{{}} , $$

in which the symbols $u^{c}$ and $y^{c}$ represent the unemployment and output gaps, respectively, the coefficients $\alpha$ and $\beta$ are in a traditional interpretation fixed unknown constants, and the term $\varepsilon$ denotes random innovations compliant with white-noise properties. The subscripts $t$ here and further indicate that respective variables relate to a particular time instance. Since Okun’s law is a non-causal (purely correlational) relationship, the arrangement of $u^{c}$ and $y^{c}$ as regressand and regressor is unimportant and can be exchanged (as is discussed, e.g., by Boďa and Považanová 2019, p. 612). Equation (1) corresponds to the gap version of Okun’s law and requires knowledge of both gap variables despite the fact that they are unobservable by its very nature.

Two issues must be addressed upon implementing the gap version. First, the gap variables $u^{c}$ and $y^{c}$ must be extracted from time series on the unemployment rate $u$ and real output $y$.^1^ Second, it transpires that the elementary static specification given in Eq. (1) is inadequate to reflect that a typical Okun relationship exhibits dynamic features and output–unemployment asymmetries. To that effect, the right-hand side of (1) is commonly extended by past values of the regressand and/or regressors and modified to incorporate possible nonlinearities.

As argued in the introduction, one approach to obtaining estimates of $u^{c}$ and $y^{c}$ is to apply an atheoretical statistical method that would perform the trend-cycle decomposition in a suitable manner. This is applied either separately to the original time series $u$ and $y$, or simultaneously to them in order to account for co-movements and co-dependence that is reasonable expect. The following exposition builds on a general notation $x$ adopted for any of the time series in question, $u$ and $y$. In a bivariate case, the notation is generalized to two time series $x^{1}$ and $x^{2}$ that actually comply with $u$ and $y$, but the order does not matter. A trend-cycle decomposition is considered for any $x$ in conformity with equation

2 $$ x_{t}^{{}} = x_{t}^{tr} + x_{t}^{c} , $$

where the superscripts $tr$ and $c$ label the trend and cycle components. The cycle component may contain seasonal variation, and it absorbs any irregularities consistent with a white noise process with zero mean. In fact, mild assumptions are demanded of the input time series $x$ (whether it be $y$ or $u$) as it may be non-stationary. Yet, the traditional account since the times of Nelson and Plosser (1982) has been that they are not trend stationary. Likewise, Hamilton (2018) argues that typical output and unemployment time series have a tendency to be difference-stationary (random-walk) processes. The three approaches, viz. the Hodrick–Prescott (HP) filter, the Hamilton (H) filter and the unobserved component model (UCM) filter, considered in the study are suited to handle both the output and unemployment dynamics. Their univariate and bivariate formulations are explicated to a necessary degree in the next three subsections. Finally, the fourth subsection gives brief comments on modelling Eq. (1) in an econometrically valid way. Distributed time effects are handled in the spirit of an auto-regressive distributed lag (ARDL) model, although by adding lagged values of $y^{c}$ only as regressors. Possible nonlinearity and asymmetric structural reactions in output–unemployment fluctuations are modelled by formulating a separate ARDL model according as a threshold is exceeded or not, in the form of a threshold ARDL (TARDL) model.

### Univariate and bivariate HP filter

In Okunian applications, the HP filter appears the most popular technique in obtaining gap variables at a small cost and can be deemed as a baseline approach (amongst others Lee 2000; Silvapulle et al. 2004; Marinkov and Geldenhuys 2007; Cevik et al. 2013; Ball et al. 2017). The cycle with the HP filter is obtained as a transitory component that remains after isolating the trend according to Eq. (2). The HP filters are often visualized as a compromise between goodness of fit and smoothness controlled by a value of the smoothing parameter whose optimal value has been intensely debated over years (e.g. Schüler 2018, pp. 3–4; Demourne et al. 2009, p. 4; Franke and Kukačka 2020, pp. 6–7). Instead of the popular objective function, the HP filter is presented in a mathematically equivalent format as a mechanic two-sided data filter with specific weights. The vector of past observed values $x_{1,...,T}^{{}} = (x_{1}^{{}} ,...,x_{T}^{{}} )^{\prime}$ is used to obtain a vector of fitted trend components $\tilde{x}_{1,...,T}^{tr} = (\tilde{x}_{1}^{tr} ,...,\tilde{x}_{T}^{tr} )^{\prime}$ and residual cyclical components $\tilde{x}_{1,...,T}^{c} = (\tilde{x}_{1}^{c} ,...,\tilde{x}_{T}^{c} )^{\prime}$ by dint of the prescriptions:

3 $$ \tilde{x}_{1,...,T}^{tr} = (I_{T}^{{}} + \lambda P^{\prime}P)^{ - 1} x_{1,...,T}^{{}} ,\quad \tilde{x}_{1,...,T}^{c} = x_{1,...,T}^{{}} - \tilde{x}_{1,...,T}^{tr} , $$

in which $\lambda$ is the penalty parameter for smoothing, $I_{T}^{{}}$ is the identity matrix of size $T$, and $P$ is a special $(T - 2) \times T$ weighting matrix induced by double differencing given by

4 $$ P = \left( {\begin{array}{cccccccc} 1 & { - 2} & 1 & 0 & \ldots & 0 & 0 & 0 \\ 0 & 1 & { - 2} & 1 & \ldots & 0 & 0 & 0 \\ \vdots & \vdots & \vdots & \vdots & \ddots & \vdots & \vdots & \vdots \\ 0 & 0 & 0 & 0 & \ldots & 1 & { - 2} & 1 \\ \end{array} } \right). $$

The popular value of $\lambda$ for quarterly data is 1,600 and is interpretable as the reciprocal value of the signal-to-noise ratio typical for US macroeconomic data (Hodrick and Prescott 1997, p. 4), and despite scathing criticism it survives. Still, at both few end-points of the available history, trend and cycle filtrates are unstable and unreliable, which may be avoided by taking into account the proposals of Kaiser and Maravall (2001, pp. 118–147). The recommended procedure is to employ a seasonally adjusted time series, identify for it an adequate ARIMA model and extend the observed series at both ends with backcasts and forecasts that are eventually discarded once the HP filter is run in the usual manner. For quarterly data, this implies backcasting and forecasting per 4 observations. The HP filter is applied here with quarterly data with the usual value of the penalty parameter with four backcast and forecast extensions.

The bivariate HP filter considered here is not the semi-structural extension of the HP filter named by Laxton and Tetlow (1992) “the multivariate filter” and by St-Amant and van Norden (1997) “the multivariate HP filter”. The extension considered by these authors adds to the optimization problem of the HP filter structural terms relating the estimated cycle to inflation dynamics (Phillips curve), unemployment fluctuations (Okun’s law) or capacity utilization (production limits). Instead, the bivariate HP filter is a non-structural extension to facilitate simultaneous filtration of product and unemployment time series by emulating the filtering mechanism of the univariate HP filter in a bivariate case. In competition to other such formulations (Reeves et al. 2000; Poloni and Sbrana 2017), this paper uses the multivariate version proposed by Dermoune et al. (2009).

In a bivariate set-up, observations of two time series are now concatenated into a $2 \times T$ vector $X_{{{1,}...{{,T}}}}^{{}} = (x_{1}^{1} ,...,x_{{{T}}}^{1} ,x_{1}^{2} ,...,x_{{{T}}}^{2} )^{\prime}$ and they are decomposed into vectors of the same size corresponding to the fitted trend $\tilde{X}_{{{1,}...{{,T}}}}^{tr} = (\tilde{x}_{1}^{1 \, tr} ,...,\tilde{x}_{{{T}}}^{1 \, tr} ,\tilde{x}_{1}^{2 \, tr} ,...,\tilde{x}_{{{T}}}^{2 \, tr} )^{\prime}$ and the fitted cycle $\tilde{X}_{{{1,}...{{,T}}}}^{c} = (\tilde{x}_{1}^{1 \, c} ,...,\tilde{x}_{{{T}}}^{1 \, c} ,\tilde{x}_{1}^{2 \, c} ,...,\tilde{x}_{{{T}}}^{2 \, c} )^{\prime}$. Dermoune et al. (2009) demonstrate how (3) can be extended in a multivariate fashion into

5 $$ \tilde{X}_{1,...,T}^{tr} = (I_{2 \times T}^{{}} + \Sigma A^{\prime}\Omega^{ - 1} A)^{ - 1} X_{1,...,T}^{{}} ,\quad \tilde{X}_{1,...,T}^{c} = X_{1,...,T}^{{}} - \tilde{X}_{1,...,T}^{tr} , $$

where $I_{2 \times T}^{{}}$ is the identity matrix of size $2 \times T$, $A$ is a $2(T - 2) \times 2T$ matrix fulfilling the role equivalent to that of $P$ in the univariate case, and $\Sigma$ and $\Omega$ are $2T \times 2T$ and ${2(T} - 2) \times 2{(T} - 2)$ matrices that together take over the role of the penalty parameter. Whereas $A$ is fixed, Dermoune et al. (2009, pp. 27–28) discuss various choices for matrices $\Sigma$ and $\Omega$. These are operationalized in such a way that they generate the same signal-to-noise ratio as the choice of 1,600 for $\lambda$ in the univariate case. First, the univariate HP filter is fitted to both time series $x^{1}$ and $x^{2}$ as described before. Then, the variances and covariances of the fitted trend and cyclical components are estimated by means of traditional moment estimators and paired appropriately with the elements of $X_{1,...,T}^{{}}$, i.e.

6 $$ \tilde{S}^{tr} = \left( {\begin{array}{ c | c} {\hat{\sigma }_{11}^{tr} I_{T - 2}^{{}} } & {\hat{\sigma }_{12}^{tr} M_{T - 2}^{{}} }_{_{}} \\ \hline {\hat{\sigma }_{12}^{tr} M_{T - 2}^{{}} } & {\hat{\sigma }_{22}^{tr} I_{T - 2}^{{}} } \\ \end{array} } \right),\quad \tilde{S}^{c} = \left( {\begin{array}{c|c} {\hat{\sigma }_{11}^{c} I_{T}^{{}} }  & {\hat{\sigma }_{12}^{c} M_{T}^{{}} }_{_{}} \\ \hline {\hat{\sigma }_{12}^{c} M_{T}^{{}} }   & {\hat{\sigma }_{22}^{c} I_{T}^{{}} } \\ \end{array} } \right), $$

where $\hat{\sigma }_{11}^{tr} ,\hat{\sigma }_{22}^{tr} ,\hat{\sigma }_{12}^{tr} ,\hat{\sigma }_{11}^{c} ,\hat{\sigma }_{22}^{c} ,\hat{\sigma }_{12}^{c}$ are the respective variance and covariance estimates for the trends and cycles of the time series $x^{1}$ and $x^{2}$, and where ${M}_{{{T}}}^{{}}$ and ${M}_{{{{T}} - {2}}}^{{}}$ are square matrices populated by ones with sizes $T$ and $T - 2$, respectively. To enforce the reciprocal signal-to-noise ratio (i.e. the cycle-to-trend ratio) at the desired level, the covariance matrices are rescaled in such a way that their average volumes make a proportion of 1,600.^2^ Whereas the matrices $\Sigma$ and $\Omega$ appearing in (5) are proportional to ${\tilde{S}}^{c}$ and ${\tilde{S}}^{tr}$, they are also demanded to satisfy that $AVol(\Sigma )/AVol(\Omega ) = 1600$. In effect, by this reasoning the following specification is applied so as to put (5) into operation:

7 $$ A = \left( {\begin{array}{*{20}c} P &\vline & {0_{(T - 2) \times T} } \\ \hline {0_{(T - 2) \times T} } &\vline & P \\ \end{array} } \right),\quad \Sigma = \tilde{S}^{c} ,\quad \Omega = \tilde{S}^{tr} \frac{{AVol(\tilde{S}^{tr} )}}{{1600 \cdot AVol(\tilde{S}^{c} )}}, $$

in which $0_{(T - 2) \times T}$ is a $(T - 2) \times T$ zero matrix.

### Univariate and bivariate UCM filter

In consequence to the extensive criticism of the HP filter (and other such similar approaches), structural time-series models of Harvey (1989) have gained popularity for two main reasons. First, they provide a better statistical representation of the dynamic process underlying economic time series. Second, they are generalizations of the HP filter in which the smoothing parameter is not chosen a priori by the analyst, but optimized with data (e.g. Harvey and Jaeger 1993, p. 233). A trend-cycle formulation of the UCM houses numerous growth typologies (e.g. Harvey 1989, pp. 45–46; Ladiray et al. 2003, pp. 39–42). Epitomes of univariate applications in Okunian analysis to obtain gap variables in the form presented here are Moosa (1997), Silvapulle et al. (2004), Huang and Lin (2006), or Marinkov and Geldenhuys (2007). Structural impositions upon the trend-cycle dynamics commenced with Clark (1987), and structurally augmented UCM filters can be found, e.g., in Chagny et al. (2004, pp. 8–10), Gusinger et al. (2018) and Čížků (2020). In line with the cited literature, the Gaussian UCM adds to the decomposition model in (2) also the seasonality component so that $x_{t}^{c} = x_{t}^{{lt{ - }c}} + x_{t}^{{{{seas}}}}$, where $x_{t}^{{lt{ - }c}}$ denotes long-term cycle (in a pure sense) and $x_{t}^{{{{seas}}}}$ represents seasonality (short-term variation). With a harmonic representation of seasonality, the UCM for quarterly data (with the length of a seasonality pattern of 4 periods) becomes

8 $$ \begin{aligned} x_{t}^{{}} & = x_{t}^{tr} + x_{t}^{lt - c} + x_{t}^{{{{seas}}}} , \\ x_{t}^{tr} & = x_{t - 1}^{tr} + \nu_{t - 1} + \varepsilon_{t}^{{{{level}}}} ,\quad \varepsilon_{t}^{{{{level}}}} \sim g(0,\delta^{{{{level}}}} ), \\ \nu_{t} & = \nu_{t - 1} + \varepsilon_{t}^{{{{slope}}}} ,\quad \varepsilon_{t}^{{{{slope}}}} \sim g(0,\delta^{{{{slope}}}} ), \\ x_{t}^{lt - c} & = x_{t - 1}^{lt - c} \cos \vartheta + x_{t - 1}^{*lt - c} \sin \vartheta + \varepsilon_{t}^{lt - c} ,\quad \varepsilon_{t}^{lt - c} \sim g(0,\delta^{lt - c} ), \\ x_{t}^{*lt - c} & = - x_{t - 1}^{lt - c} \sin \vartheta + x_{t - 1}^{*lt - c} \cos \vartheta + \varepsilon_{t}^{*lt - c} ,\quad \varepsilon_{t}^{*lt - c} \sim g(0,\delta^{lt - c} ), \\ x_{t}^{{{{seas}}}} & = s_{1,t} + s_{2,t} , \\ s_{j,t} & = s_{j,t - 1} \cos (\pi j) + s_{j,t - 1}^{*} \sin (\pi j) + \varepsilon_{j,t}^{{{{seas}}}} ,\quad \varepsilon_{j,t}^{{{{seas}}}} \sim g(0,\delta^{{{{seas}}}} ),\quad j \in \{ 1,2\} , \\ s_{j,t}^{*} & = - s_{j,t - 1} \sin (\pi j) + s_{j,t - 1}^{{}} \sin (\pi j) + \varepsilon_{j,t}^{{{*seas}}} ,\quad \varepsilon_{j,t}^{{*{{seas}}}} \sim g(0,\delta^{{{{seas}}}} ),\quad j \in \{ 1,2\} , \\ \end{aligned} $$

in which all disturbances $\varepsilon_{{t}}^{{{{\vphantom{slope}level}}}}$, $\varepsilon_{{t}}^{{{{slope}}}}$, $\varepsilon_{{t}}^{{{\vphantom{slope}lt{ - }c}}}$, $\varepsilon_{{t}}^{{{\vphantom{slope}*lt{ - }c}}}$, $\varepsilon_{{j,t}}^{{{{\vphantom{slope}seas}}}}$, $\varepsilon_{{j,t}}^{{{{\vphantom{slope}*seas}}}}$ are independent and the parameters to estimate are the unknown variances $\delta_{{}}^{{{{level}}}}$, $\delta_{{}}^{{{{slope}}}}$, $\delta^{{lt{ - }c}}$, $\delta^{{{{seas}}}}$ (all positive) and the cyclical frequency $\vartheta$ in radians (so that $0 \le \vartheta \le \pi$). These parameters are by default estimated by maximum likelihood (ML), and the components coming out of the decomposition are estimated by Kalman filtering and smoothing (see, e.g., Harvey 1989, pp. 100ff).

In the cited studies, exploring Okun’s law filtering via the univariate UCM is applied separately for output and unemployment series. The decomposition described by (8) can be easily adapted to both time series at a time by using Eq. (8) for each of them and by allowing contemporaneous correlation between disturbances of the cyclical (long-term and short-term seasonal) components. In effect, the bivariate UCM explains the dynamics of both input time series $y$ or $u$ by Eq. (8) with two additional parameters introduced: correlation between innovations of $y$ or $u$ in their long-term cycle equations ($\varepsilon_{t}^{{lt{ - }c}}$ and $\varepsilon_{t}^{{*lt{ - }c}}$) and the seasonal variation equations ($\varepsilon_{j,t}^{{{{seas}}}}$ and $\varepsilon_{j,t}^{{{*seas}}}$). All other innovations are assumed independent. A similar stance was taken by Cuaresma (2003) whose bivariate system was less restrictive as it allowed correlated effects underlying the “trend” dynamics ($\varepsilon_{t}^{{{{level}}}}$) and the “slope” dynamics ($\varepsilon_{t}^{{{{slope}}}}$). The imposition of the same frequency $\vartheta$ for $y$ or $u$ unifies cyclical variations in output and unemployment and presumes that business cycles manifest themselves equally in these time series. Ladiray et al. (2003, pp. 59–60) illustrate how a multivariate UCM can be simplified to tackle the multiplicity of innovations and their correlations.

### Univariate and bivariate H filter

A simple response to the numerous drawbacks of the HP filter is the filter by Hamilton (2018) who suggested constructing a simple $h$-period-ahead linear forecasting rule by regressing the current value of the series, $x_{t}^{{}}$, on $r$ past values shifted at least $h$ periods backwards, $x_{t - h}^{{}} ,$ $x_{t - h - 1}^{{}} ,...,x_{t - h - r + 1}^{{}}$, in order to obtain trend estimates. For quarterly macroeconomic data, Hamilton (2018) recommends $h = 8$ and $r = 4$. That is, the application of the H filter for a quarterly time series requires running a linear regression using the available history of data in the form

9 $$ x_{t}^{{}} = \gamma_{0} + \gamma_{1} x_{t - 8}^{{}} + ... + \gamma_{4} x_{t - 11}^{{}} + \zeta_{t} , $$

where $\zeta_{t}$ is a white-noise disturbance term and $\gamma_{0} ,\gamma_{1} ,...,\gamma_{4}$ are regression parameters estimated by ordinary least squares. This produces an estimate of the trend component, $\tilde{x}_{t}^{tr} = \tilde{x}_{t}^{{}}$, and accordingly an estimate of the cyclical component as a residual, $\tilde{x}_{t}^{c} = x_{t}^{{}} - \tilde{x}_{t}^{tr}$. In the face of the ambitious endeavour, a sequence of studies investigating the performance of the H filter indicates that this solution is not a panacea (e.g. Schüler 2018; Phillips and Shi 2019; Hodrick 2020; Franke and Kukačka 2020). To the best knowledge of the authors, except Arčabić and Olson (2019) there has been no relevant application of the H filter in relation to Okun’s law.

A multivariate extension of the H filter is obvious, and it is a seemingly unrelated regression (SUR) system, in which both time series at issue, $y$ or $u$, are regressed according to formula (9), but allowing for contemporaneous correlations between their disturbance terms. The system of two equations can be estimated by estimated generalized least squares without any special protocol (see, e.g., Judge et al. 1985, pp. 466ff), and the bivariate predictions are used then as trend estimates, and the residuals as cycle estimates.

### ARDL and TARDL model

Having denoted the traditional backshift operator as *L* and by equipping Eq. (1) with short-term dynamics, the gap version of Okun’s law may be more adequately stated as

10 $$ u_{t}^{c} \, = \, a + {\rm B}_{q} (L)y_{t}^{c} + \sigma e_{t} , $$

where $\, a$ is an intercept, ${\rm B}(L)$ stands for a standard auto-regressive polynomial with real coefficients defined as ${\text{B}}_{q} (L) = b_{1} + b_{2} L^{1} + ... + b_{q + 1} L^{q}$. The parameter $q$ in the polynomial measures the length of time distributed effects (with $q \ge 0$). The last term $\sigma e_{t}$ consists of standard deviation $\sigma$ (such that $\sigma > 0$) and white noise $e_{t}$ with zero mean and unit standard deviation. The representation given by (10) is an ARDL(0,*q*) model, in which (auto-regressive) effects induced by the regressand are not present and *q *is the length of time effects induced by the regressor. Whereas the coefficients in ${\text{B}}_{q} (L)$ are instantaneous multipliers, the long-run multiplier defined as $\beta = {\text{B}}_{q} (1) = \sum_{k} b_{k}$ fulfils the role of a long-run multiplier mapping influence of $y_{t}^{c}$ upon $u_{t}^{c}$. Ordinary least squares are an unbiased and consistent estimator for $a$ and the coefficients in ${\rm B}_{q} (L)$, and an additional Gaussian assumption for $e_{t}$ justifies statistical inference. A concise textbook exposition of the ARDL model and its estimation is Greene (2003, pp. 571–579).

As is documented in the literature, the intensity of the output–unemployment relationship varies with the business cycle, which injects nonlinearity into the equation, no matter whether it is considered in the form of (1) or (10). Empirical economics, reassured by common wisdom, discovered that such asymmetries are typically ascribable to a particular threshold variable $z_{{\text{t}}}$ that gives rise to different regression equations in relation to the value of one or more thresholds that divide the real axis. It is also a convenient method to tackle possible structural changes that can be tracked to the threshold variable. For example, the analysed period, 2003/Q1–2021/Q4, covers several strenuous economic epochs for the G7 countries or the world economy, such as the Great Recession and Global Financial Crisis of 2007–2009, the US housing bubble of 2006–2012, the European sovereign debt crisis in 2010. In addition, it includes the recent COVID-19 Recession. Typically one cut-off point $\theta$ suffices, and two regimes are distinguished: a regime for small values of $z_{{\text{t}}}$ (say, $z_{t} \le \theta$) and a regime for high values (say, $z_{t} > \theta$). This observation gave rise to the theory of threshold auto-regression, and a number of applications have emerged (Hansen 2011). Threshold auto-regressions are described in sufficient detail, e.g., in Zivot and Wang (2006, pp. 662–678), and the blending of threshold modelling with ARDL models is owing to Greenwood-Nimmo et al. (2011) and Shin et al. (2013). Threshold ARDL (TARDL) models were applied in modelling Okun’s law by Silvapulle et al. (2004), Marinkov and Geldenhuys (2007), Tang and Bethencourt (2017), wherein the threshold variable $z_{t}$ was represented by $y_{t}^{c}$ and the threshold itself was set to zero. Cuaresma (2003) allowed the threshold-free and searched for an appropriate value in a vein similar to Boďa et al. (2015). Lee (2000) augmented the basic static equation to incorporate asymmetries around zero, although with a different arrangement of the regressand and regressor. Unlike the other cited ARDL studies, auto-regressive effects are in this paper disregarded, and unemployment fluctuations are explained only by time-distributed effects of output fluctuations. The threshold variable $z_{t}$ is associated here with the output gap and expressed as an output gap accumulated over the last four quarters on a sliding basis. Whereas the output gap for a quarter measures by how much real GDP deviated from potential in that particular quarter, the threshold variable adopted here captures the size of this deviation for the running year. Owing to the use of logarithimized real GDP for $y$, the definition of $z_{t}$ is

11 $$ z_{1}^{{}} = y_{1}^{c} {,}\quad z_{2}^{{}} = y_{1}^{c} + y_{2}^{c} {,}\quad z_{3}^{{}} = y_{1}^{c} + y_{2}^{c} + y_{3}^{c} {,}\quad z_{t}^{{}} = y_{t}^{c} + y_{t - 1}^{c} + y_{t - 2}^{c} + y_{t - 3,}^{c} \, \quad {\text{for}}\quad t \ge {4,} $$

where the definition of the three first values reflects the end-of-sample problem.

A somewhat generic two-regime TARDL model can be represented as:

12 $$ \begin{gathered} u_{t}^{c} {{ = a}}_{{}}^{1} + {\rm B}_{{{{q}}_{1} }}^{1} {{(L)}}y_{t}^{c} + {\upsigma }_{{}}^{1} {\text{e}}_{{\text{t}}} ,\quad {\text{for}}\quad z_{{\text{t}}} \le {\theta }\;\;{\text{(regime 1),}} \\ u_{t}^{c} {{ = a}}_{{}}^{2} + {\rm B}_{{{{q}}_{2} }}^{2} {{(L)}}y_{t}^{c} + {\upsigma }_{{}}^{2} {\text{e}}_{{\text{t}}} ,\quad {\text{for}}\quad z_{{\text{t}}} > {\theta }\;\;{\text{(regime 2),}} \\ \end{gathered} $$

where the upper superscripts 1 and 2 identify the regime to which the parameters ${\rm B}_{q_i}^{i} (L)$_,_ $a^{i}$ and ${\upsigma }^{i}$ answer. For $i = 1$, it is in a down-regime with $z_{t} \le \theta$, whereas for $i = 2$, it is in an up-regime with $z_{t} > \theta$. The lag length is typically set identical in different regimes, i.e. $q_{1} = q_{2}$. In an application of model (12), the threshold variable $z_{{\text{t}}}$ is known, but in finding an adequate cut-off value $\theta$, several approaches have been debated in the literature. Following Tsay (1989) and Granger and Teräsvirta (1993, pp. 114–115), the lag lengths $q = q_{1} = q_{2}$ are chosen, for example, by a suitable model building strategy so that $e_{t}$ complies with white-noise assumptions, and then, a stepwise search is performed over the interval of values attained by $z_{t}$. To assure that the threshold specification in (12) is sensible in comparison with a nonlinear specification in (10), a linearity test is required. The testing may be carried by the Hansen bootstrap test formulated by Hansen (1996, 1997) originally threshold auto-regressive models with two regimes and exposited by Zivot and Wang (2006, pp. 662–663, 669–671).

## Data and results

The analysis was applied to quarterly macroeconomic data of the seven G7 countries: Canada (CA), France (FR), Germany (DE), Italy (IT), Japan (JP), the United Kingdom (GB) and the United States (US). The codes in parentheses are later applied in charts for identification. Data were sourced from the OECD database as of 25 April 2022, and all were available seasonally adjusted.^3^ Data on real GDP prior to the logarithmization were stated in the national currency (as chain volume estimates with different reference years), and unemployment was measured as a percentage rate with respect to total labour force. A total of 124 observations for each time series were effectively available spanning the period of 31 years from 1991/Q1 to 2021/Q4. The period at issue contains various economically critical moments, such as the Great Recession or the COVID-19 Recession that might have altered the structural rigidity of Okun’s law, which gives grounds for using threshold regression. The former recessionary event happened soon after the midpoint of the data frame, whereas the latter occurred at its end. Albeit a longer history of data is available, the selected span of three decades for most of the G7 countries represents an economically and politically coherent period for this sort of an analysis of output and unemployment fluctuations. The start of the time frame concurs with the unification of Germany, the stabilization of economic and international relations after the end of the Cold War or the advent of the Internet.

**Fig. 1:**
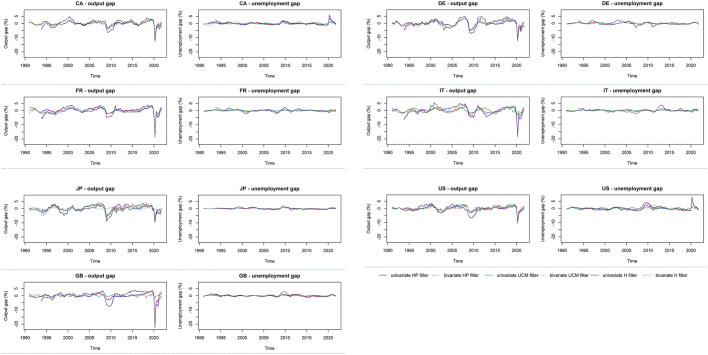
Trajectories of the estimated gap variables

**Fig. 2:**
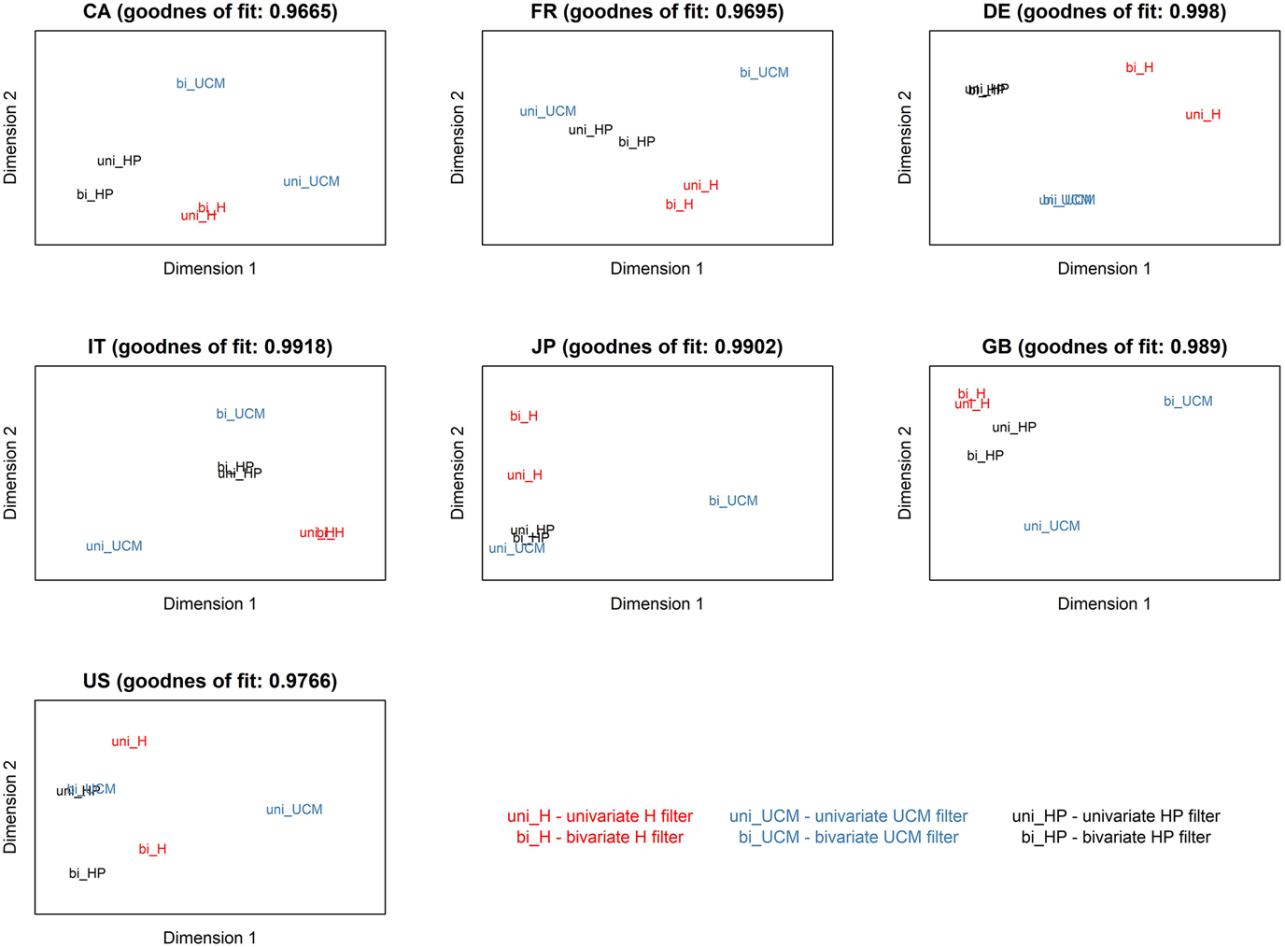
Similarity of the filtering approaches in terms of estimated Okun coefficients on maps

Program R (R Core Team 2019) served the analysis with some of its extra packages, ${\mathtt{CCA}},\, {\mathtt{dynlm}}, \,{\mathtt{forecast}}, \,{\mathtt{KFAS}},\, {\mathtt{mFilter}}, \,{\mathtt{neverhpfilter}}, \,{\mathtt{systemfit}},\, {\mathtt{urca}}, \,{\mathtt{vars}}, \,{\text{and}}\, {\mathtt{zoo}}$. Absent procedures (e.g. for the bivariate H filter or TARDL estimation) were programmed by the authors. Numeric results are for their extensity relegated to Appendixes 1, 2 and 3, and graphical displays are organized within the text as Figs. 1 and 2. Estimated gaps are stated in percentages or percentage points.

For the HP and UCM filter, the estimated gaps span the entire period of 124 quarters, but for the H filter owing to its construction the first 11 observations were lost to obtain filtrates. Hence, for both the univariate and bivariate variant of the H filter there are only 113 quarters of gap estimates, running from 1993/Q4 to 2021/Q4. This also has an impact upon the estimation of Okun equations and results in a loss of degrees of freedom.

Figure 1 exhibits different trajectories of the estimated gap variables arising from the six different filtering methodologies. In most cases, they tend to give visually similar indications of oscillatory patterns, and the trajectories agree in the majority of cases on the timing of conjunctural variations and their amplitude. For instance, the intense response of both output and unemployment to the COVID-19 pandemic in 2020 is perfectly visible from sharp declines in the output gap and sharp upswings of the unemployment gap. Fluctuations revealed in unemployment gaps are comparatively milder in comparison with those manifested in output gaps. Furthermore, output gaps seem more erratic, and it is apparently for them that the filtering approaches are in most disagreement. A thorough inspection of the paths in the individual charts confirms that differences are present also between the univariate and bivariate forms of the same filtering technique. The UCM filters tend to identify regular (almost ideally trigonometric) cycles, which is discernible well for Italy, Japan and the UK ($y^{c}$).

In spite of seeming visual congruence, the difference in the estimated gap variables is revealed in the basic statistical summary put forward in Appendix 1 and as a matter of fact also in the correlation report in Appendix 2. The displayed statistics in Appendix 1 indicate differences in both location and dispersion that are detectable in most cases. That said, inspecting differences between the gap estimates by individual quarters, almost identical estimates are found with the univariate and bivariate UCM filters for Germany ($y^{c}$, $u^{c}$), Japan ($u^{c}$), or France and the UK ($y^{c}$), whilst only slight differences are between the univariate and bivariate H and HP filters for France and the UK ($u^{c}$) or between the univariate and bivariate HP filter for Germany, Italy and Japan ($u^{c}$), The most marked heterogeneity in the estimated gaps is detected between both versions of the UCM filter and both versions of the H filter in the case of the UK, or between the univariate UCM filter and bivariate H filter for Italy and Japan ($y^{c}$). Methodologically correct econometric estimation of Okunian Eqs. (10) and (12) requires that both gap variables are stationary or co-integrated. To this end, Appendix 1 also reports the results for unit-root testing using two well-established procedures. The augmented Dickey–Fuller (ADF) test posits a unit root in the null hypothesis as opposed to the Kwiatkowski–Phillips–Schmidt–Shin (KPSS) test that has a unit root in the alternative hypothesis. The combined use of the ADF and KPSS test is a recommended procedure to check unit root non-stationarity (e.g. Schlitzer 1995, 1996). The details on the adopted configuration of the tests are placed into the note beneath the table in Appendix 1. For most gap estimates, stationarity is confirmed unanimously by both unit-root tests, and only exceptions are a few cases with the univariate and/or bivariate UCM or H filter. Specifically, doubts possibly arise for six gap estimates for Germany, Italy and Japan ($y^{c}$, $u^{c}$), even though in four cases the KPSS tests indicate stationarity. Furthermore, informal means of stationarity inspection do not validate a presence of a unit root. It also must be noted that the HP filter is capable of producing non-stationary filtrates in typical sample sizes (e.g. Sakaraya and de Jong 2020; Phillips and Jin 2020). In contrast, with the H filter this issue in an empirical setting is not appreciated yet despite the assurance of Hamilton (2018) that for a broad range of processes the extracted cycle is stationary. The summary of correlation coefficients displayed in Appendix 2 may help assess the consonance of gap estimates yielded by different filtering techniques and their agreement with an inverse output–unemployment relationship prescribed by Okun’s law. Means, standard deviations and ranges reported for each country in Appendix 2 were computed from the 15 pairwise correlation coefficients for all six output and unemployment gap estimates and from the 36 pairwise correlation coefficients resulting from matching six output and six unemployment gap estimates. It should be noted that maximum values of correlation coefficients 1.000 are recorded only in consequence of their rounding. Although the paths of the identified gap variables exhibited in Fig. 1 testify to a high level of visual co-movement, the correlation report shows that the congruence in many cases is not so strong and the estimated gap variables may be fairly distinct, if (positively) correlated. This is especially manifested in the minimums and means of the correlation coefficients for both gap variables.

The massive tabular report in Appendix 3 displays the results of one-regime and two-regime threshold regressions using an ARDL(0,*q*) framework. The lag length* q* is identified for Eq. (10) using the Schwarz information criterion in order to establish a parsimonious representation and is applied unanimously for both Eqs. (10) and (12) allowing a delay of 4 quarters at most. For one-regime regressions, the table reports (i) two Okun coefficients $\beta$ established as long-run multipliers by summing either all instantaneous Okun coefficients (i.e. $\sum_{k} {b}_{k}$) or only those significant at a 0.05 level of significance (i.e. $\sum_{{{\text{p-value}}(b_{k} ) \le 0.05}} {b}_{k}$), and (ii) coefficients of determination (adjusted R squared) as goodness-of-fit measures. For threshold regressions, the table organizes this information appropriately for both regimes (“d” for a down-regime, and “u” for an up-regime) alongside the estimated threshold used in classifying the regimes and the numbers of observations in both regimes. Eventually, the last columns of the table report the results of the Hansen nonlinearity test performed with 2,000 bootstrap replications. Threshold regression is statistically supported only if the null hypothesis of no asymmetric effects is rejected.

As it happens, the results for each country are heterogeneous, albeit a greater variety of results plagues the two-regime threshold regressions. Nonetheless, regarding the one-regime regressions, in all G7 countries except the UK the long-run multipliers $\beta$, regardless of their significance, are found all with the right (negative) signs, so they subscribe to the validity of Okun’s law. For the UK, two long-run multipliers $\beta$ drop to zero once the criterion of 0.05 significance is taken into account, whilst others retain their negative sign. The said issue with insignificance is found only for both versions of the UCM filter. In spite of the uniformity in signs, the Okun long-run multipliers $\beta$ even in one-regime regressions are fairly distant. For Canada, the maximum difference between a pair of long-run multipliers is 0.530, for France it is only 0.143, for Germany this difference amounts to 0.168, for Italy it is only 0.076, for Japan it is 0.072, for the United Kingdom the difference makes 0.146, whereas for the USA the discrepancy is largest at 0.806. Also the length of time delay is extremely differentiated between the filtering methods for the same country. The only exception is the UK, for which only contemporaneous influence ($q = 0$) of the output gap on the unemployment gap is detected for each filtering method. For Italy and the USA, the output gap might exert only contemporaneous influence ($q = 0$) or its influence could emerge from one more quarter in the past ($q = 1$). For other countries, the effects might be between contemporaneous or two quarters delayed (for France and Germany), between contemporaneous or three quarters delayed (for Canada) or even they could stretch up to four quarters back (for Japan). Finally, the estimated one-regime regressions also differ in terms of their goodness of fit, even for the same country, ranging from poor (say, adjusted R squared smaller than 0.10) to fairly good (say, adjusted R squared larger than 0.80). A simple exploratory analysis^4^ of R-squared values not only acknowledges the apparent fact that the adjusted R-squared measure generally improves with increasing the lag length $q$, but also reveals the impact of the filtering method. The estimated Okun one-regime regressions display the comparatively best R-squared values for the H filter and the worst for the UCM filter. To this pattern, the dimensionality of the filter does not matter.

A much more varied picture is discovered when examining whether a nonlinear TARDL model is a more apt description of the output–unemployment relationship than a linear ARDL model. The F statistic for testing nonlinearity is evaluated at a 0.05 level of significance and is found significant 22 times. Threshold regression is supported for France unanimously with all the six filtering methods, whereas for Canada, Italy and the USA threshold regression is statistically preferred in the case of four filtering methods (for Canada and USA, all but the bivariate HP filter and univariate UCM filter, and for Italy all but the two variants of the UCM filter). For other countries, threshold nonlinearity is supported with three filtering methods for the UK (specifically, both variants of the UCM filter and the bivariate H filter), and with one filtering method for Germany (the univariate H filter). Only for Japan, one-regime linear regression is preferable over two-regime threshold regression regardless of the filtering method. Apparently, there is no uniformity or regularity.

All other results related to threshold regressions may be correctly considered only if nonlinearity is detected. Also the estimated threshold values, constructed as the trailing annual output gap through the definition in (11), are subject to immense variation. For example, the six identified threshold values for France vary between −12.975 and 4.109%, which is the only country for which they are found with changing sings. For other G7 countries, threshold values with statistically significant threshold regressions have all negative signs. In some cases, particularly when implemented with either variant of the H filter, threshold values are fairly high by all standards. This holds particularly for France, Germany, Italy or the UK where for the H filter the threshold values range between −15.656 and −11.134. The threshold variable is constructed as a running total of four consecutive quarterly output gaps, which makes it estimate-specific and linked with a particular estimate of the output gap. In consequence, these threshold values are not directly comparable and may, and obviously do, lead to diverse classifications of quarterly observations into down-regimes and up-regimes across the six filtering methods. The distinct measurements of the threshold variable associated with different estimates of the output gap affect threshold values, which also passes into different divisions of observations into the down-regimes and up-regimes. For example, for France the four strictly negative down-regimes with negative thresholds (for the HP and H filters) are populated by 13 to 71 observations, bud the other two down-regimes with positive thresholds (for the UCM filters) count 82 to 86 observations. For other countries, all down-regimes are separated with negative thresholds; these are for Canada, Germany and Italy in the range from 13 to 33 observations, whilst for the UK and the USA they range between 13 and 44 observations.

Significant threshold regressions are also at odds in characterizing the down-regime and up-regime responses of unemployment fluctuations to output fluctuations that translate into regime-specific long-run multipliers $\beta$. For Canada, Okun long-run multipliers $\beta$ in down-regimes and up-regimes are all negative, but in three cases the sensitivity of unemployment to output is found sharper in the down-regime (the univariate HP filter and both versions of the H filter), whereas in one case this sensitivity is lessened in the down-regime is found less sensitive (the bivariate UCM filter). For France, only with one filtering method both Okun coefficients are negative (the bivariate UCM filter), and five cases of significant threshold regressions are identified with an insignificant or positive relationship between output and unemployment fluctuations. For Germany and Italy, all significant threshold regressions have either an insignificant or positive long-run multiplier $\beta$ regardless of the filtering method. In contrast, for the USA all long-run coefficients $\beta$ are correctly negative and the heightened responsiveness is established for down-regimes.

It seems that it might be advisable to avoid using a threshold variable formed as a model-specific estimate since the inescapable uncertainty underpinning the model also passes into the threshold variable. In this present context, the annual output growth rate (defined possibly as a running total of four consecutive differences of quarterly logarithmized real GDP) could be preferable over the trailing annual output gap (operationalized as a running total of four consecutive estimated quarterly output gaps) despite the fact that the latter is more closely connected with the notion of cyclical fluctuations.

The USA can serve as an example of the diversity of the findings. For the univariate H filter, the F statistic is (convincingly) insignificant at a 0.05 level of significance and the Hansen test points to a presence of nonlinearity in Okun’s relationship. The threshold is optimized at −0.46%, which means that for the period between 1991/Q1 and 2021/Q4 when US real GDP was under potential and deviated downwards from potential GDP by more than −0.46% in the last four quarters (a down-regime), the Okun coefficient was estimated on average at −0.620. Conversely, when in the examined period the deviation of US real GDP from potential was more than −0.46% (an up-regime), the estimate of the Okun coefficient is then −0.445. This is, in essence, only a negligible difference. Nonetheless, for the bivariate HP filter, the hypothesis of linearity is not rejected, and the results for one-regime regression apply. The Okun coefficient is estimated uniformly at −0.877, which is not even an average of the other two regime-specific Okun coefficients. In contrast to the two-regime regression with the output gap found exerting a one-quarter delayed effect upon the unemployment gap, the relationship in the one-regime regression is found merely contemporaneous. By going over the results in Appendix 3, it is obvious that the findings are at variance and multifarious.

Finally, the similarity and disparity of the filtering methods can be visualized in several ways. One simple approach is through multidimensional scaling (MDS) performed with respect to six attributes separately for each country. Countries are represented by a sextet of coordinates represented by long-run multipliers $\beta$ reported in the table of Appendix 3, i.e. the Okun coefficients in one-regime regressions (all and significant only) as well as those in two-regime regressions for down-regimes and up-regimes (all and significant only). Classical (metric) MDS described, for example, in Everitt (2005, pp. 93–96) takes the six coordinates and replaces them by two coordinates so that the Euclidean distances of objects represented here by different filtering methods are retained at a minimum loss of information. For each country, a map showing relative positions of the filtering methods is drawn and presented in Fig. 2 alongside information on the quality of fit. Goodness-of-fit metrics are all above 96.65% and point to usually an excellent fit. A configuration similar (in material respect identical) to the one displayed in the maps of Fig. 2 is obtained if in place of the six long-run multipliers a different set of criteria is considered, viz. threshold values, lag lengths, and only significant long-run multipliers (one-regime, down-regime and up-regime). Albeit the filtering techniques do create clusters, these are not consistent across countries. Usually, the results indicated by the univariate and bivariate variants of a filtering method are alike and positioned in close vicinity. This is true for 6 countries in the case of the HP filter and 4 countries in the case of the H filter. The similarity between the univariate and bivariate implementation of the UCM filter is shown only for one country. Aside from the similarity of the results for the HP and H filter, in the majority of cases it is difficult to find a systematic pattern.

## Discussion

In spite of its role in economic policy modelling and forecasting, Okun’s law may be deemed as a simplistic empirical relationship or correlation that has been continually proven unstable in applied research, which is especially owing to different set-ups and modelling choices. Nonetheless, the advantage of the gap version is that it helps to stabilize the relationship predicted by Okun’s law and that reduces cross-country heterogeneity in empirical estimates. Whereas unemployment arises as a mismatch between employment (labour demand) and labour force participation (labour supply), both these driving forces are linked to output, and these links vary over the business cycle (Sögner and Stiassny 2002). When output and unemployment gaps (the gap version) are used in place of period-on-period changes (the difference version), some or most of temporal variation is filtered out and purer effects come out. In consequence, a practical question is which method of estimating output and unemployment gaps should be preferred. A vast body of literature is committed towards this question. Some authors seek criteria that a trend-cycle decomposition method should satisfy so that its estimates of gap variables may be viewed as reasonable and relevant to applied economics. A sound method should produce estimates of output (and possibly unemployment) gaps that are stable in the face of ex post revisions and plausible in the light of new data (Celov and Network of Independent Fiscal Institutions 2020, p. 15). Cuerpo et al. (2018, pp. 276–278) formulate three principles for optimality of an output gap estimation method, according to which an adequate method should balance economic soundness, statistical goodness and transparency. As a matter of fact, by these principles, the data-driven approaches applied in this paper perform comparatively poorly, but their advantage is simplicity and prevalence in academic research. Nelson (2008) favours using estimated output gaps to forecast future output growth and comparing different methods by their forecast accuracy. This approach has been generally accepted, and forecast accuracy is not only evaluated in conjunction with output growth predictions (e.g. Kamber et al. 2018), but also with inflation predictions when embedded in the Phillips curve (e.g. Furlanetto et al. 2020). Although these standards are fairly handy in a context of finding a reasonable method to measure the output (and possibly unemployment) gap, they are not directly helpful in identifying a reasonable estimate of Okun's law. The reason being, Okun’s law per se is an empirically uncovered relationship that is believed to exist between output and unemployment fluctuations, which themselves are not directly measurable as being derived from the unobservable potential output and natural rate of unemployment. Like these fluctuations, a true Okun relation is a fiction on account of a lack of consensus amongst economists on the rationale of Okun’s law. A straightforward implication is the fact that there is no universal Okunian equation, but an equation with a rather loose arrangement of the sides and plenty of methods that have been utilized or developed to estimate Okun's law. Another point is that an Okun coefficient encapsulates no normative aspect; it merely captures the compensating (and certainly not causal) co-movement between fluctuations in production and unemployment. It is inevitably associated with the manner how these fluctuations, or gap variables, are conceptualized and estimated. Hence, no yardstick exists to which its credibility can be measured or compared to the extent that gap variables themselves are estimated in a credible fashion. Okun's law is a useful instrument of economic policy and is a link in theoretical or empirical models simply because it has been found to work. For these reasons, the plausibility and stability of gap variables are not a guarantee that Okun's law may or must be estimated properly.

**Fig. 3:**
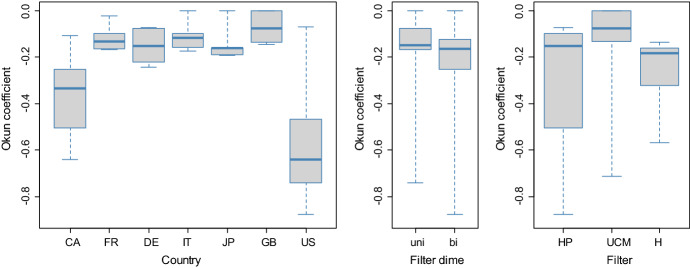
Variation in Okun coefficients. *Note*: Okun coefficients displayed in the box-plots are long-run multipliers identified by summing short-run multipliers significant at a 0.05 level of significance

As Okun’s law has been frequently estimated with each of the data-driven methods considered in the case study (e.g. Cuaresma 2003; Kim et al. 2020; Donayre 2022), this practice is unlikely to change in the future. Although the veracity of estimates of Okun coefficients cannot be reasonably judged, some insights still can be said on the effect of initial modelling choices on the results. One-regime Okun coefficients may be affected by a presence of country effects, the application of a particular filtering method characterized by dimensionality and choice of the filter, lag length and the existence of threshold nonlinearity, in which case two sets of Okun coefficients should apply. Appendix 4 reports the results of two regressions, in which long-run Okun multipliers $\beta$ are regressed on the set of these candidate predictors. These multipliers and all predictors are compiled from Appendix 3, and the regression analysis considers both full summative coefficients and coefficients trimmed at a 0.05 significance level. Only country effects and filter type are detected significant at this level of significance. Filter dimensionality, threshold nonlinearity or lag-length does not seem to impact upon the magnitude of Okun coefficients. Save perturbations in distant decimal places, the regression outputs in Appendix 4 do not change with the removal of these insignificant predictors, which would otherwise give the models found optimal in regard to the Schwarz information criterion. Yet, these simplified models are not reported. No interaction between filter type and filter dimensionality is established, and neither are other interactions between the predictors. Figure 3 confronts long-run Okun multipliers $\beta$ trimmed for significance differentiated by country, filter dimensionality and filter type. Country effects are discernible, and so is the fact that the UCM filter tends to yield more dampened values of Okun coefficients. Nonetheless, except different heterogeneity, there seems no difference in the magnitude of Okun coefficients between the HP and H filters. Filter dimension does not exhibit a systematic effect. In this respect, Ačabić and Olson (2019) compare for 20 OECD countries Okun coefficients estimated with the use of the HP filter with those estimated with the use of the H filter so as to conclude that the former approach tends to yield Okun coefficients of a smaller magnitude. Here this kind of statement would suit the coefficients produced in conjunction with the UCM filter. For down-regime and up-regime Okun coefficients, this comparative analysis is complicated by the fact that threshold values vary with particular estimates of the output gap and that there are only 22 effective observations available when threshold linearity is indicated by the Hansen test. An analysis of this sort for down-regime and up-regime coefficients is thus avoided.

Okun's law enjoys the status of a stylized fact, and its full rejection is extremely rare (e.g. de la Foneijne 2014). However, what is debated in addition to asymmetries and nonlinearities is instability over time and time variance (e.g. Lee 2000; Sögner and Stiassny 2002; Meyer and Tasci 2012; Michail 2019). Some other concern may be related to the effect of possible structural breaks in the business cycle as there is abundant evidence that threshold models may fail to differentiate between innate nonlinearity and nonlinear patterns induced by structural breaks (e.g. Koop and Potter 2001) or gap estimates may themselves be affected by structural breaks (e.g. Perron and Wada 2009; Coibion et al. 2018). Nonetheless, the latter concern is dispelled by the outcome of the unit-root testing procedure whose results are reported as part of Appendix 1. The dual utilization of the ADF and KPSS test indicates strongly that except two cases for Germany, one case for Italy and three cases of Japan all estimated gap variables are stationary, and in these three exceptional cases the status is otherwise uncertain. In consequence, the concern of distortions due to a presence of structural breaks is not substantiated. As far as the issue of stability is indicated, the estimated one-regime ARDL models are applied also to four subperiods that arise by slicing the time frame of 31 years into four equal parts spanning 7 years 3 quarters and counting nominally 31 quarterly observations. Subperiod I goes from 1991/Q1 to 1998/Q3, Subperiod II runs from 1998/Q4 to 2006/Q2, Subperiod III ranges from 2006/Q3 to 2014/Q1, whereas Subperiod IV ranges from 2014/Q2 to 2021/Q4. Note that Subperiod III begins with the Great Recession and Subperiod IV ends with the COVID-19 Recession. For each subperiod, one-regime ARDL models with lag lengths as reported in Appendix 3 are fitted by using a shorter span of data, and Okun coefficients are determined as long-run multipliers $\beta$. Appendix 5 reports Okun coefficients for each country compared to Okun coefficients for the whole period. To make study of subperiod differences easier, the coefficients are drawn in the form of line graphs equipped with additional information on the effective number of observations and adjusted R-squared values. The graphs display Okun coefficients to which the criterion of a 0.05 significance level is applied, which is the reason why in some cases Okun coefficients are zero. The graphs also report a measure of variability of subperiod Okun coefficients labelled as RMSE (root mean square error) and computed as the square root of the average square difference of subperiod Okun coefficients from the whole-period Okun coefficient.

The trajectories of subperiod Okun coefficients in Appendix 5 reveal that Okun's law is found insignificant mostly in Subperiods I and II when there were no strenuous economic conditions.^5^ A worse fit is also generally obtained for Subperiods I and II when subperiod R-squared values are confronted with the whole period R-squared value, albeit there is no apparent pattern.^6^ In some cases, extremely poor fits are signalled by negative values, and these happen especially for the UCM filter in one of its implementation for 12 subperiod Okun coefficients (for Canada, France, Germany, the UK and the USA). One such case of a negative subperiod R squared is observed for the univariate HP filter (for the UK).

The line graphs in Appendix 5 reveal that there are fluctuating patterns in the magnitude of Okun coefficients over the four subperiods that signify time variance of Okun coefficients. Since whole-period Okun coefficients are conceptually mere weighted averages of subperiod coefficients, systematic deviations of subperiod Okun coefficients from whole-period Okun coefficients can be suggestive of time variance of Okun’s law. Admittedly, the evidence of the varying correlation of output–unemployment fluctuations is only a collateral finding, but there is striking synchronicity between the diverse gap estimation methods for a country, but asynchrony between the G7 countries themselves. For Canada, Okun's law is of a smaller magnitude, or weaker in intensity, typically in Subperiods II and III (6 and 4 cases), for France it is in Subperiods I and IV (5 and 6 cases), for Germany in Subperiods I, III and IV (5, 6 and 6 cases), for Italy for Subperiods I, III and IV (5, 4 and 6 cases), for Japan in Subperiods I, III and IV (5, 6 and 5 cases), for the UK in Subperiods II and IV (4 and 4 cases), and the USA in Subperiods I and II (6 and 6 cases). The tendencies towards less intense Okun's law are discernible in the graphs of Appendix 5 as points lying above the dashed horizontal lines anchoring the values of whole period Okun coefficients. They also encompass situations of insignificant subperiod coefficients, which are concentrated in Subperiods I and II. The trajectories of Okun coefficients do not reveal that they could be affected by the Great Recession or the recent pandemic economic downturn.

**Fig. 4:**
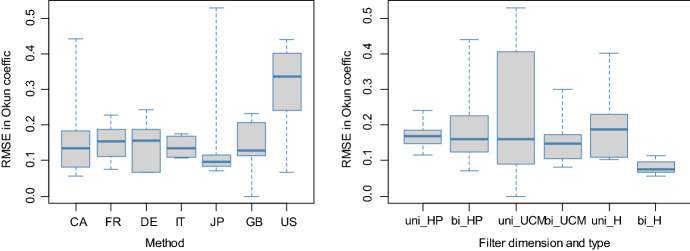
Variation in Okun coefficients for Subperiods I, II, III and IV. *Note*: Okun coefficients displayed in the box-plots are long-run multipliers identified by summing short-run multipliers significant at a 0.05 level of significance

The variability in subperiod Okun coefficients as measured by RMSE is comparatively high with a value greater than 0.19 in five cases, namely with the univariate UCM filter for Canada, the univariate HP filter for France, the bivariate HP filter for Germany, the bivariate UCM filter for Japan, and with the bivariate HP filter for the USA. Appendix 4 reports the results of two regressions of RMSE upon country effects, filter dimension and filter type. One regression is fitted for RMSE coming from full Okun coefficients, and the other regression is fitted for RMSE arising from Okun coefficients trimmed for a 0.05 significance level. Figure 4 shows box plots comparing RMSE answering to significant Okun coefficients for countries and filters and is suggestive of two notable deviations from the uniform pattern of RMSE values, although not supported by the regression result. First, for the USA the filtering methods appear to be in comparatively higher disagreement in how they capture subperiod invariance of Okun's law. Second, gap estimates produced by the bivariate Hamilton filter appear to yield the comparatively least subperiod heterogeneity of Okun coefficients.

The identified heterogeneity of the results coming from diverse filtering specifications is very comparable to that indicated by Perman et al. (2015) in their meta-analysis of 269 estimated Okun coefficients compiled from 28 studies. It should be evaluated on two tiers. The first tier is not outwardly linked with Okun’s law. Output and unemployment gap estimates are heavily utilized in macroeconomic analyses and economic planning in measuring the state and prospects of aggregate economic activity. On the one hand, it is at first glance evident that structural approaches utilizing information on connections between different areas of an economy should be superior to purely statistical approaches. On the other hand, the former approaches dominate practical economic analysis and planning in policy institutions that are more ready to deploy large-scale models and have better access to data), whereas the latter are typical for academic research (that suffers from unavoidable limitations). Both Fig. 1 and Appendix 1 reveal that estimated gap variables may differ, and they do. On the one hand, most cases displayed in Fig. 1 reveal synchronicity and concord in phases and amplitudes. On the other hand, there are still differences in some cases and the summaries in Appendixes 1 and 2 prove that the differences may be substantial. Without a priori knowledge of the (economically structural, not merely statistical) data-generating process of macroeconomic time series, any gap output or unemployment estimate is a guess at best, even if its credibility may be enhanced by using various approaches at a time and by seeking their agreement. There also remain issues of laying down the universal definition of the output gap (e.g. Kiley 2013, pp. 9–10). A flourishing research front is structural New-Keynesian models in which output gap is defined as the difference between actual output and its flexible-price counterfactual indicated by a model (e.g. Gálí et al. 2012), which implies a structural specification of Okun’s law in gaps.

**Fig. 5:**
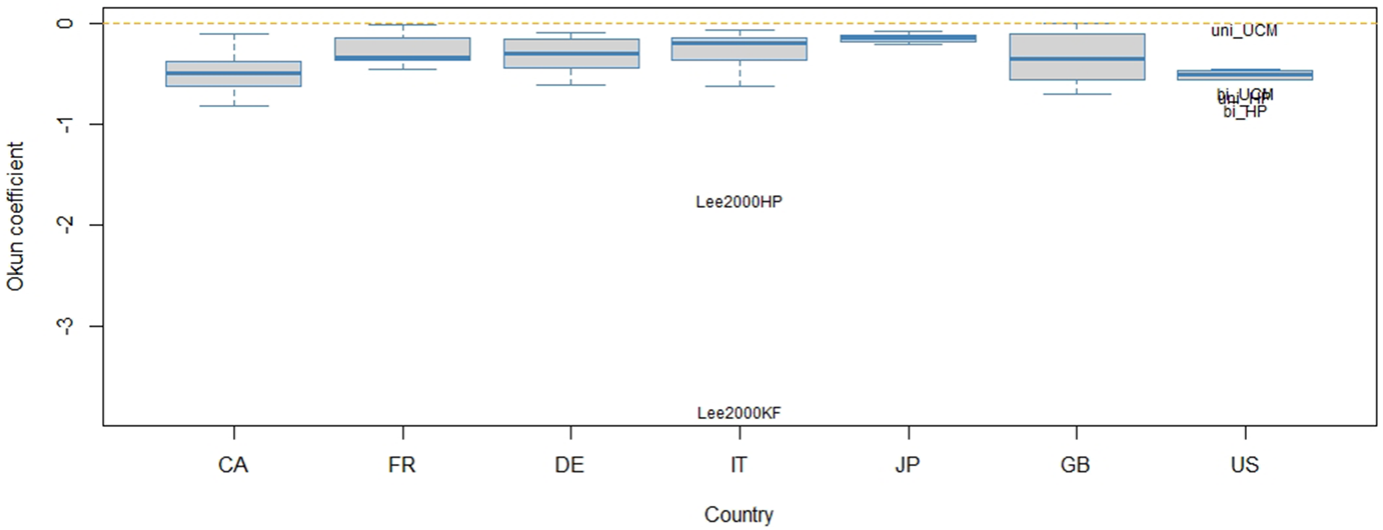
Comparison of one-regime Okun coefficients with other studies. *Note*: Please study the note under the table in Appendix 6. “Lee2000HP” and “Lee2000KF” refer to the results of Lee (2000) where he estimated gap variables by the HP filter and Kalman filter, respectively. The labels “uni_HP”, “bi_HP”, “uni_UCM” and “bi_UCM” refer to the univariate and bivariate HP filter as well as the univariate and bivariate UCM filter

The second tier is how different specifications affect estimation of the output–unemployment relationship represented by Okun’s law that was handled in this study in the spirit of distributed lag modelling to account for time distributed effects. It is also common wisdom that Okun’s law may be well nonlinear due to its asymmetry over the business cycle (Silvapulle et al. 2004; Nebot et al. 2019), although this contention seems not to be fully embraced by professional forecasters in the G7 countries (Pierdzioch et al. 2011). Irrespective of what truth is in the existence of asymmetry, a combination of gap variables may indicate that such Okun’s law may possess nonlinear features over the business cycles. It is alarming that the results are not far from being unified in this regard. Conditional on a particular approach to estimating gap variables in combination with data, there is no rule whether linear one-regime regression or two-regime threshold regression may be pinpointed as more descriptive and trusted. Inevitably, the results are dependent on the choice of filtering approaches, the adoption of a modelling framework and the specification of a threshold variable. Nonetheless, even this specific set-up makes the point. It goes without saying that also other approaches to estimating output and unemployment gaps would reaffirm a varied picture, and higher diversity would be revealed with other threshold variables. That said, the currently utilized threshold variable measuring the deviation of real GDP from its potential in the past four quarters is not contemporaneous and safely does the job of mapping Okun’s law to the business cycle. The adopted (T)ARDL modelling framework has an advantage that preserves some comparability of the present coefficients with those of other studies, although they depart in techniques to constructing gap variables and differ in both data and time frame. To exploit this benefit, Appendix 6 compares the Okun coefficients reported in Appendix 3 with those established by some other studies. Any such comparison must be done with caution, and it is advisable to read the explanatory notes beneath the tables in Appendix 6. It is now apparent that when the Okun coefficients sprouting here from different filtering techniques are compared, they do not seem altogether atypically heterogeneous. To the contrary, they fit relatively well amongst values compiled from the extant research in spite of the different methodologies as is also discernible in Fig. 5 that shows one-regime Okun coefficients in the form of box-plots sketched for countries. Of course, to all intents and purposes, the intervals implied by the box (the middle 50% values) and the whiskers (the range) are fairly wide, and hence unsatisfactory for considerate and targeted economic policy or forecasting. Furthermore, it makes no sense to arrange countries in terms of the strength with which Okun’s law manifests itself.

It might be interesting to perform an analysis similar to the meta-analysis of Perman et al. (2015) who assumed that there exists for all time periods and for all countries a unique, yet identical, value of the Okun coefficient and examined published results for a presence of publication bias. The authors of this study are reluctant to accept such a unifying assumption and to cast all results into a funnel plot or test explicitly for funnel asymmetry.

## Conclusion

The present research cautions against naïve interpretations of Okun coefficients as every particular value is a legacy of numerous choices to the point until this value is determined by a suitable estimation method. There are absolutely no standards to judge credibility of one value against another, which may be eased by considering several approaches at a time and comparing the results. The outlined comparative procedure would inevitably lead to several competitive values whose dispersion cannot be warranted small or whose likelihood cannot be assessed a priori without an exact knowledge of the true data generating process for macroeconomic data. One may hypothesize structural relationships between different parts of an economy and make premises about aggregate behaviour of economic agents. Still, there is a problem how to choose between several possibilities. An instrumentalist’s solution is difficult to apply in real time when a value for the Okun coefficient is needed. This is especially seen in the fact that not only the Okun coefficients supplied by different methods may be inconveniently scattered over a large interval of values (as is displayed in Fig. 5 chiefly for the UK), but they may produce conflicting results concerning a possible presence of nonlinear responses of unemployment to the business cycle. Nonetheless, the filtering methods considered in this paper are consistent in the sense that they all point to similar time-varying patterns of Okun's law and are fairly synchronized as to indicating fluctuations in the magnitude of Okun coefficients. They also tend to measure similar country differences in the responsiveness of output–unemployment fluctuations despite the fact that the UCM filter is found to produce gap estimates that lead to relatively smaller Okun coefficients than the HP or H filters do.

It must be admitted that the six filtering approaches applied for quarterly data of the G7 countries can shed little light on what one can expect. The finding that gap estimates may differ from method to method is not novel, and neither is the fact that statistical approaches without a structural input have a limited trend-cycle decomposition potential (St-Amand and van Norden 1997, pp. 2, 34; Grant and Chan 2017, p. 114). Yet, they are unlikely to be superseded in academic research by much more sophisticated techniques with structural insights whether it be in connection with Okunian analysis or for other purposes. The problem is the said absence of standards for assessing estimates of Okun coefficients. Albeit Okun (1962) came up with regression analysis in a very elementary set-up, since then numerous procedures have been devised to help address issues associated with estimation of the output–unemployment relationship. It is difficult to assert that such-and-such a method is preferable and that a given value of the Okun coefficient is plausible. There are approaches that model time-varying features and non-constancy of the Okun coefficients (e.g. Huang and Lin 2006, 2008) or that treat asymmetry via Markov regime-switching models (e.g. Cevik et al. 2013), and these give other insights or supply answers to specific research questions. A possible avenue might be to demand that an Okunian analysis does not only provide values of the Okun coefficient estimated by means of a sound and generally accepted method, but these values are accompanied by confidence intervals (perhaps set by a defendable bootstrap method). Any similar set of standards would have to be tested before generally adopted. Nonetheless, the intention of this paper is not bridge this gap.

## Data Availability

Available on request.

## Appendix 1: Descriptive statistics and stationarity reports for the estimated gap variables

**Table:**

	Output gap						Unemployment gap					
	HP filter		UCM filter		H filter		HP filter		UCM filter		H filter	
	Univariate	Bivariate	Univariate	Bivariate	Univariate	Bivariate	Univariate	Bivariate	Univariate	Bivariate	Univariate	Bivariate
*Canada*												
Mean^†)^	−0.058	−0.011	−0.093	−0.170	0.000	0.000	0.032	0.054	0.006	−0.003	0.000	0.000
Standard deviation^†)^	1.577	1.329	1.602	1.926	2.592	2.609	0.807	0.901	0.343	0.835	1.099	1.110
Minimum^†)^	−11.637	−10.911	−11.826	−12.872	−12.919	−12.743	−1.348	−1.400	−0.818	−1.487	−1.406	−1.361
Median^†)^	−0.009	0.066	−0.028	0.057	0.370	0.373	−0.055	0.011	−0.004	−0.068	−0.254	−0.268
Maximum^†)^	2.694	2.907	2.339	2.412	4.867	4.509	5.684	5.838	0.982	3.873	6.085	6.250
ADF test statistic^‡)^	−5.402^**^	−6.702^**^	−5.137^**^	−4.345^**^	−3.670^**^	−3.533^**^	−4.992^**^	−4.440^**^	−4.333^**^	−4.535^**^	−4.151^**^	−4.052^**^
KPSS test statistic^‡)^	0.035^ ns^	0.023^ ns^	0.057^ ns^	0.100^ ns^	0.081^ ns^	0.084^ ns^	0.035^ ns^	0.058^ ns^	0.047^ ns^	0.055^ ns^	0.111^ ns^	0.086^ ns^
Suggested status^‡)^	Stationary	Stationary	Stationary	Stationary	Stationary	Stationary	Stationary	Stationary	Stationary	Stationary	Stationary	Stationary
*France*												
Mean^†)^	−0.051	−0.062	−0.166	−0.168	0.000	0.000	0.004	0.008	0.007	0.001	0.000	0.000
Standard deviation^†)^	1.995	1.917	2.433	2.428	2.820	2.855	0.424	0.482	0.256	0.463	0.966	0.971
Minimum^†)^	−17.797	−17.377	−19.087	−19.115	−18.221	−17.877	−1.273	−1.363	−0.368	−1.181	−2.299	−2.377
Median^†)^	−0.125	−0.111	−0.214	−0.211	0.277	0.400	0.024	0.054	−0.001	−0.022	0.007	0.005
Maximum^†)^	3.320	3.408	3.318	3.312	3.980	4.155	0.783	0.847	0.365	0.973	2.293	2.267
ADF test statistic^‡)^	−5.796^**^	−6.183^**^	−4.451^**^	−4.452^**^	−4.328^**^	−4.185^**^	−3.656^**^	−3.332^**^	−17.836^**^	−3.133^*^	−3.518^**^	−3.384^**^
KPSS test statistic^‡)^	0.036^ ns^	0.027^ ns^	0.210^ ns^	0.211^ ns^	0.103^ ns^	0.152^ ns^	0.040^ ns^	0.064^ ns^	0.027^ ns^	0.045^ ns^	0.294^ ns^	0.250^ ns^
Suggested status^‡)^	Stationary	Stationary	Stationary	Stationary	Stationary	Stationary	Stationary	Stationary	Stationary	Stationary	Stationary	Stationary
*Germany*												
Mean^†)^	−0.048	−0.028	−0.133	−0.127	0.000	0.000	0.008	0.007	0.014	0.014	0.000	0.000
Standard deviation^†)^	1.780	1.692	2.128	2.128	3.035	3.144	0.485	0.521	0.328	0.328	1.059	1.066
Minimum^†)^	−10.712	−10.253	−11.499	−11.494	−12.296	−11.009	−1.079	−1.207	−0.464	−0.464	−3.123	−2.918
Median^†)^	−0.070	0.014	−0.121	−0.116	0.213	−0.230	0.031	0.004	0.038	0.038	−0.094	−0.075
Maximum^†)^	3.670	3.554	3.546	3.552	4.902	5.126	1.349	1.481	0.465	0.465	2.326	2.348
ADF test statistic^‡)^	−4.425^**^	−4.708^**^	−3.559^**^	−3.559^**^	−3.211^*^	−3.184^*^	−5.363^**^	−5.124^**^	−51.167^**^	−51.167^**^	−3.526^**^	−3.202^**^
KPSS test statistic^‡)^	0.037^ ns^	0.024^ ns^	0.073^ ns^	0.073^ ns^	0.105^ ns^	0.401^•^	0.046^ ns^	0.048^ ns^	0.030^ ns^	0.030^ ns^	0.513^*^	0.565^*^
Suggested status^‡)^	Stationary	Stationary	Stationary	Stationary	Stationary	Stationary	Stationary	Stationary	Stationary	Stationary	Uncertain	Uncertain
*Italy*												
Mean^†)^	−0.073	−0.094	−0.065	−0.222	0.000	0.000	−0.010	−0.014	−0.014	−0.026	0.000	0.000
Standard deviation^†)^	2.128	2.139	1.236	2.329	3.268	3.296	0.466	0.456	0.229	0.536	1.088	1.096
Minimum^†)^	−16.883	−16.927	−3.043	−16.773	−18.776	−18.652	−1.230	−1.227	−0.388	−1.149	−2.430	−2.438
Median^†)^	−0.034	−0.030	0.006	−0.054	0.471	0.514	−0.010	−0.017	−0.014	−0.019	−0.002	−0.026
Maximum^†)^	3.326	3.356	2.160	4.115	5.589	4.961	1.131	1.113	0.357	1.381	3.808	3.815
ADF test statistic^‡)^	−5.080^**^	−5.048^**^	−3.033^*^	−4.089^**^	−3.617^**^	−3.611^**^	−3.527^**^	−3.573^**^	−4.275^**^	−2.588^•^	−3.281^*^	−3.075^*^
KPSS test statistic^‡)^	0.033^ ns^	0.035^ ns^	0.033^ ns^	0.164^ ns^	0.239^ ns^	0.206^ ns^	0.060^ ns^	0.054^ ns^	0.028^ ns^	0.085^ ns^	0.178^ ns^	0.178^ ns^
Suggested status^‡)^	Stationary	Stationary	Stationary	Stationary	Stationary	Stationary	Stationary	Stationary	Stationary	Uncertain	Stationary	Stationary
*Japan*												
Mean^†)^	−0.067	−0.031	−0.138	−0.001	0.000	0.000	−0.001	−0.002	0.001	0.001	0.000	0.000
Standard deviation^†)^	1.609	1.532	2.182	0.366	2.749	2.919	0.259	0.268	0.381	0.381	0.579	0.619
Minimum^†)^	−8.284	−7.858	−9.592	−0.711	−9.736	−8.308	−0.623	−0.651	−0.861	−0.861	−1.310	−1.259
Median^†)^	−0.012	0.054	−0.126	0.003	1.032	0.572	−0.014	−0.011	−0.059	−0.058	−0.073	0.024
Maximum^†)^	2.925	2.874	4.093	0.654	3.322	4.081	0.885	0.896	0.961	0.961	1.762	1.737
ADF test statistic^‡)^	−4.208^**^	−4.486^**^	−2.908^*^	−2.679^•^	−3.090^*^	−2.925^*^	−3.804^**^	−3.722^**^	−2.701^•^	−2.701^•^	−3.022^*^	−3.090^*^
KPSS test statistic^‡)^	0.047^ ns^	0.032^ ns^	0.137^ ns^	0.048^ ns^	0.142^ ns^	0.524^ ns^	0.048^ ns^	0.046^ ns^	0.074^ ns^	0.073^ ns^	0.610^*^	0.673^*^
Suggested status^‡)^	Stationary	Stationary	Stationary	Uncertain	Stationary	Uncertain	Stationary	Stationary	Stationary	Stationary	Uncertain	Uncertain
*UK*												
Mean^†)^	−0.089	−0.089	0.003	0.003	0.000	0.000	0.038	0.042	0.021	0.004	0.000	0.000
Standard deviation^†)^	2.358	2.238	0.625	0.624	3.401	3.423	0.429	0.536	0.273	0.161	0.829	0.834
Minimum^†)^	−20.932	−20.315	−1.865	−1.833	−22.661	−22.337	−1.000	−1.044	−0.676	−0.225	−1.426	−1.440
Median^†)^	0.034	0.041	0.040	0.040	0.879	1.002	−0.043	−0.085	0.001	0.037	−0.088	−0.142
Maximum^†)^	3.447	3.688	1.268	1.232	3.724	3.784	1.230	1.447	0.966	0.229	2.783	2.800
ADF test statistic^‡)^	−5.532^**^	−5.959^**^	−7.164^**^	−7.177^**^	−3.772^**^	−3.665^**^	−3.752^**^	−3.197^*^	−5.281^**^	−29.157^**^	−3.822^**^	−4.069^**^
KPSS test statistic^‡)^	0.042^ ns^	0.036^ ns^	0.026^ ns^	0.025^ ns^	0.089^ ns^	0.096^ ns^	0.130^ ns^	0.153^ ns^	0.039^ ns^	0.015^ ns^	0.168^ ns^	0.163^ ns^
Suggested status^‡)^	Stationary	Stationary	Stationary	Stationary	Stationary	Stationary	Stationary	Stationary	Stationary	Stationary	Stationary	Stationary
*USA*												
Mean^†)^	−0.051	0.007	−0.140	−0.036	0.000	0.000	0.036	0.022	0.012	0.037	0.000	0.000
Standard deviation^†)^	1.382	1.031	1.732	1.534	2.353	2.462	1.067	1.384	0.399	1.095	1.481	1.520
Minimum^†)^	−9.670	−8.633	−10.504	−9.755	−9.849	−10.576	−1.544	−1.809	−0.833	−1.686	−1.736	−1.604
Median^†)^	−0.034	0.053	−0.169	0.021	0.518	0.260	−0.071	−0.159	−0.014	0.015	−0.420	−0.486
Maximum^†)^	2.361	2.342	2.987	2.627	3.427	4.010	7.770	7.819	1.190	7.228	8.011	8.259
ADF test statistic^‡)^	−4.558^**^	−6.561^**^	−3.567^**^	−4.053^**^	−3.290^*^	−2.982^*^	−4.844^**^	−3.572^**^	−4.670^**^	−4.138^**^	−3.901^**^	−3.688^**^
KPSS test statistic^‡)^	0.042^ ns^	0.023^ ns^	0.117^ ns^	0.052^ ns^	0.086^ ns^	0.194^ ns^	0.042^ ns^	0.079^ ns^	0.016^ ns^	0.061^ ns^	0.285^ ns^	0.231^ ns^
Suggested status^‡)^	Stationary	Stationary	Stationary	Stationary	Stationary	Stationary	Stationary	Stationary	Stationary	Stationary	Stationary	Stationary

*Legend*: Significance labels displayed at computed statistics convey the following meaning: ^**^ for p-values ≤ 0.01, ^*^ for p-values ≤ 0.05, ^•^ for p-values ≤ 0.10, and ^ns^ for p-values > 0.10

^†)^ Reported descriptive statistics are expressed as percentages (for output gaps) or percentage points (for unemployment gaps) per annum. ^‡)^ Since unit-root and stationarity tests are notorious for their low power (e.g. Cochrane 1991; Kočenda and Černý 2015, p. 72ff), both a unit root test and a stationarity test are applied simultaneously. Whilst the former is the drift version of the augmented Dickey–Fuller test (“ADF test”) formulated by Said and Dickey (1984), the latter is the “mu” version of the Kwiatkowski–Phillips–Schmidt–Shin test (“KPSS test”) developed by Kwiatkowski et al. (1992). The testing strategy is dual: the ADF test is required to reject the null hypothesis of a unit root, whereas the KPSS test is required not to reject the null of its stationarity. This is in step with the approach of Schlitzer (1996) or Kočenda and Černý (2015, p. 73) who advocate simultaneous testing. Computed test statistics are confronted with asymptotic critical values assembled from Dickey and Fuller (1981), Hamilton (1994) and Kwiatkowski et al. (1992). The testing is carried out at a 0.05 level of significance. A conflicting conclusion of the tests is indicated as “uncertain”

## Appendix 2: Correlation between the estimated gap variables

**Table:**

	Different output gap estimates between themselves				Different unemployment gap estimates between themselves				Estimated output versus unemployment gaps			
	Mean	Standard deviation	Minimum	Maximum	Mean	Standard deviation	Minimum	Maximum	Mean	Standard deviation	Minimum	Maximum
Canada	0.873	0.087	0.715	0.994	0.689	0.217	0.434	0.991	−0.735	0.160	−0.911	−0.445
France	0.888	0.059	0.816	1.000	0.663	0.245	0.227	0.996	−0.236	0.088	−0.446	−0.107
Germany	0.893	0.066	0.801	1.000	0.533	0.272	0.213	1.000	−0.509	0.073	−0.718	−0.420
Italy	0.773	0.199	0.475	0.999	0.619	0.221	0.324	0.996	−0.224	0.144	−0.525	0.044
Japan	0.637	0.318	0.197	0.991	0.785	0.168	0.602	1.000	−0.517	0.214	−0.847	−0.027
UK	0.561	0.330	0.199	1.000	0.626	0.196	0.331	0.994	−0.290	0.176	−0.597	−0.017
USA	0.815	0.115	0.611	0.986	0.718	0.248	0.320	0.975	−0.734	0.188	−0.958	−0.308

## Appendix 3: Estimated models

**Table:**

	Lag length	Two-regime threshold regression						One-regime regression			Nonlinearity test^†)^	
		Threshold	# observations	β		R-squared		β		R-squared	F statistic	P-value
				Significant^‡)^	All	Regimes	Model	Significant^‡)^	All			
*Canada*												
Univariate HP filter	1	−4.08	d: 20	d: −0.615	d: −0.615	d: 0.918	0.866	−0.504	−0.504	0.847	18.199^**^	0.003
			u: 104	u: −0.446	u: −0.446	u: 0.710						
Bivariate HP filter	1	−1.982	d: 28	d: −0.637	d: −0.637	d: 0.733	0.712	−0.639	−0.639	0.693	6.087^ ns^	0.249
			u: 96	u: −0.366	u: −0.491	u: 0.430						
Univariate UCM filter	0	0.521	d: 70	d: −0.082	d: −0.082	d: 0.142	0.248	−0.108	−0.108	0.243	1.952 ns	0.61
			u: 54	u: −0.115	u: −0.115	u: 0.092						
Bivariate UCM filter	3	−3.448	d: 33	d: −0.348	d: −0.345	d: 0.582	0.482	−0.252	−0.263	0.305	39.032^***^	0
			u: 91	u: −0.482	u: −0.512	u: 0.401						
Univariate H filter	1	−6.802	d: 24	d: −0.407	d: −0.474	d: 0.745	0.813	−0.322	−0.381	0.776	27.426^***^	0
			u: 89	u: −0.164	u: −0.261	u: 0.475						
Bivariate H filter	2	−8.944	d: 21	d: −0.229	d: −0.459	d: 0.736	0.852	−0.35	−0.376	0.796	48.618^***^	0
			u: 92	u: −0.209	u: −0.263	u: 0.785						
*France*												
Univariate HP filter	1	−0.46	d: 65	d: 0.059	d: 0.031	d: 0.195	0.497	−0.099	−0.09	0.183	41.115^***^	0
			u: 59	u: −0.195	u: −0.156	u: 0.133						
Bivariate HP filter	2	−0.086	d: 71	d: 0.064	d: 0.050	d: 0.112	0.454	−0.139	−0.133	0.179	40.609^***^	0
			u: 53	u: −0.257	u: −0.259	u: 0.190						
Univariate UCM filter	0	4.109	d: 86	d: 0.000	d: 0.013	d: 0.004	0.241	−0.024	−0.024	0.038	36.344^***^	0
			u: 38	u: −0.103	u: −0.103	u: 0.167						
Bivariate UCM filter	2	3.639	d: 82	d: −0.048	d: −0.048	d: 0.229	0.409	−0.124	−0.098	0.249	23.026^**^	0.004
			u: 42	u: −0.535	u: −0.495	u: 0.385						
Univariate H filter	1	−12.83	d: 14	d: 0.000	d: 0.185	d: 0.138	0.488	−0.167	−0.177	0.246	41.979^***^	0
			u: 99	u: −0.360	u: −0.372	u: 0.533						
Bivariate H filter	1	−12.98	d: 13	d: 0.000	d: 0.218	d: 0.154	0.495	−0.165	−0.194	0.292	29.355^***^	0
			u: 100	u: −0.301	u: −0.339	u: 0.518						
*Germany*												
Univariate HP filter	0	0.374	d: 72	d: −0.056	d: −0.056	d: 0.048	0.536	−0.149	−0.149	0.29	3.876^ ns^	0.254
			u: 52	u: 0.000	u: −0.048	u: 0.020						
Bivariate HP filter	0	0.556	d: 78	d: −0.064	d: −0.064	d: 0.044	0.441	−0.153	−0.153	0.233	4.403^ ns^	0.194
			u: 46	u: 0.000	u: −0.049	u: 0.008						
Univariate UCM filter	2	−1.175	d: 55	d: −0.049	d: −0.124	d: 0.251	0.418	−0.075	−0.1	0.318	5.698^ ns^	0.596
			u: 69	u: −0.168	u: −0.206	u: 0.529						
Bivariate UCM filter	2	−1.153	d: 55	d: −0.049	d: −0.124	d: 0.251	0.417	−0.075	−0.1	0.318	5.631^ ns^	0.612
			u: 69	u: −0.168	u: −0.207	u: 0.529						
Univariate H filter	0	−11.13	d: 19	d: 0.000	d: −0.005	d: −0.058	0.538	−0.222	−0.222	0.396	37.382^***^	0
			u: 94	u: −0.364	u: −0.364	u: 0.556						
Bivariate H filter	0	−6.25	d: 39	d: 0.000	d: −0.008	d: −0.026	0.596	−0.244	−0.244	0.507	2.987 ns	0.535
			u: 74	u: −0.249	u: −0.249	u: 0.454						
*Italy*												
Univariate HP filter	1	−4.722	d: 24	d: 0.093	d: 0.097	d: 0.352	0.436	−0.104	−0.09	0.174	22.004^***^	0.001
			u: 100	u: −0.198	u: −0.193	u: 0.312						
Bivariate HP filter	1	−4.821	d: 24	d: 0.094	d: 0.101	d: 0.352	0.424	−0.098	−0.083	0.159	22.543^***^	0.001
			u: 100	u: −0.194	u: −0.185	u: 0.310						
Univariate UCM filter	0	−3.368	d: 111	d: 0.000	d: 0.083	d: −0.012	0.132	−0.132	−0.132	0.104	0.201^ ns^	1
			u: 13	u: 0.000	u: −0.084	u: 0.016						
Bivariate UCM filter	1	−9.221	d: 13	d: 0.114	d: 0.111	d: 0.684	0.438	−0.133	−0.11	0.248	1.862^ ns^	0.901
			u: 111	u: −0.181	u: −0.130	u: 0.201						
Univariate H filter	0	−15.66	d: 13	d: 0.128	d: 0.128	d: 0.294	0.436	−0.158	−0.158	0.21	37.764^***^	0
			u: 100	u: −0.298	u: −0.298	u: 0.446						
Bivariate H filter	0	−14.11	d: 15	d: 0.138	d: 0.138	d: 0.268	0.502	−0.174	−0.174	0.262	37.628^***^	0
			u: 98	u: −0.307	u: −0.307	u: 0.506						
*Japan*												
Univariate HP filter	4	−4.936	d: 16	d: −0.198	d: −0.245	d: 0.651	0.786	−0.163	−0.183	0.759	15.568^ ns^	0.16
			u: 108	u: −0.146	u: −0.212	u: 0.738						
Bivariate HP filter	3	−4.587	d: 14	d: −0.207	d: −0.247	d: 0.701	0.764	−0.19	−0.19	0.732	16.103	0.064
			u: 110	u: −0.156	u: −0.224	u: 0.713						
Univariate UCM filter	2	−2.833	d: 49	d: −0.125	d: −0.147	d: 0.375	0.72	−0.16	−0.16	0.701	10.686^ ns^	0.164
			u: 75	u: −0.160	u: −0.222	u: 0.740						
Bivariate UCM filter	0	−1.726	d: 13	d: 0.000	d: −1.277	d: 0.181	−0.007	−0.122	−0.122	0.204	10.107^ ns^	1
			u: 111	u: 0.000	u: −0.110	u: 0.001						
Univariate H filter	1	−12.9	d: 13	d: −0.239	d: −0.202	d: 0.286	0.559	−0.163	−0.163	0.537	9.809^ ns^	0.129
			u: 100	u: −0.116	u: −0.149	u: 0.373						
Bivariate H filter	1	5.315	d: 64	d: −0.186	d: −0.186	d: 0.588	0.786	−0.194	−0.194	0.774	1.970^ ns^	0.987
			u: 49	u: −0.077	u: −0.072	u: 0.104						
*UK*												
Univariate HP filter	0	−2.087	d: 42	d: 0.000	d: 0.007	d: −0.020	0.611	−0.074	−0.074	0.161	18.682^***^	0
			u: 82	u: −0.124	u: −0.124	u: 0.266						
Bivariate HP filter	0	−3.048	d: 19	d: 0.037	d: 0.037	d: 0.168	0.47	−0.076	−0.076	0.092	14.394^***^	0.001
			u: 105	u: −0.194	u: −0.194	u: 0.205						
Univariate UCM filter	0	−1.342	d: 36	d: 0.000	d: 0.103	d: −0.002	0.015	0	−0.06	0.058	1.298^ ns^	0.965
			u: 88	u: 0.000	u: −0.080	u: 0.025						
Bivariate UCM filter	0	2.012	d: 112	d: 0.000	d: −0.015	d: −0.006	−0.004	0	−0.004	0.046	3.693^ ns^	0.542
			u: 12	u: 0.291	u: 0.291	u: 0.309						
Univariate H filter	0	−14.83	d: 13	d: 0.000	d: 0.024	d: −0.075	0.465	−0.137	−0.137	0.302	8.788^*^	0.029
			u: 100	u: −0.213	u: −0.213	u: 0.364						
Bivariate H filter	0	−16.8	d: 13	d: 0.000	d: 0.019	d: −0.082	0.488	−0.146	−0.146	0.345	7.487	0.069
			u: 100	u: −0.212	u: −0.212	u: 0.384						
*USA*												
Univariate HP filter	1	−5.35	d: 13	d: −0.800	d: −0.941	d: 0.907	0.875	−0.741	−0.741	0.83	48.870^***^	0
			u: 111	u: −0.623	u: −0.623	u: 0.708						
Bivariate HP filter	0	−2.092	d: 13	d: −0.726	d: −0.726	d: 0.761	0.525	−0.877	−0.877	0.417	4.172^ ns^	0.184
			u: 111	u: −0.544	u: −0.544	u: 0.100						
Univariate UCM filter	0	2.218	d: 87	d: −0.101	d: −0.101	d: 0.135	0.14	−0.071	−0.071	0.081	5.417^ ns^	0.141
			u: 37	u: 0.131	u: 0.131	u: 0.084						
Bivariate UCM filter	1	−5.985	d: 13	d: −0.711	d: −0.802	d: 0.930	0.95	−0.713	−0.713	0.926	65.215^***^	0
			u: 111	u: −0.629	u: −0.629	u: 0.910						
Univariate H filter	1	−0.456	d: 44	d: −0.620	d: −0.755	d: 0.795	0.776	−0.467	−0.547	0.706	17.289^***^	0.005
			u: 69	u: −0.445	u: −0.587	u: 0.473						
Bivariate H filter	0	−0.999	d: 35	d: −0.679	d: −0.679	d: 0.890	0.901	−0.568	−0.568	0.843	67.678^***^	0
			u: 78	u: −0.244	u: −0.244	u: 0.379						

*Legend*: Tags “d” and “u” denote, respectively, the downturn and upturn regimes with an economic growth rate below or above the threshold. Significance labels displayed at computed statistics convey the following meaning: ^***^ for p-values ≤ 0.001, ^**^ for p-values ≤ 0.01, ^*^ for p-values ≤ 0.05, ^•^ for p-values ≤ 0.10, and ^ns^ for p-values > 0.10

^†)^ The testing for nonlinearity is based upon the test developed by Hansen (1996, 1997) originally for TAR(2) models, i.e. threshold auto-regressive models with two regimes. In this context, the null hypothesis holds that there is no threshold nonlinearity and the estimated output gap depends linearly on current and lagged values of the unemployment gap. The p-value is estimated by a bootstrap procedure with a total of 2,000 simulations. ^‡)^ Long-run Okun coefficients are identified from estimated short-run multipliers significant at a 0.05 level of significance

## Appendix 4: Impact of regression configuration upon Okun coefficients and their variability

**Table:** Regression of long-run multipliers *β* upon configuration of filters and other factors

Response variable	Intercept	Country effects^‡)^						Bivariate filter^‡)^	Filter type^‡)^		Lag length^‡)^	Non-linearity^‡)^	R squared	# observations
		FR	DE	IT	JP	GB	US		UCM	H				
All β^†)^	−0.389^**^	0.261^***^	0.209^*^	0.270^**^	0.207^•^	0.288^**^	−0.212^ ns^	−0.062^ ns^	0.142^*^	0.020^ ns^	−0.005^ ns^	−0.009^ ns^	0.695	42
Significant β^†)^	−0.383^**^	0.242^***^	0.203^*^	0.244^**^	0.221^*^	0.279^**^	−0.217^ ns^	−0.065^ ns^	0.161^*^	0.034^ ns^	−0.008^ ns^	−0.001^ ns^	0.684	42

*Legend*: Significance labels displayed at computed statistics convey the following meaning: ^***^ for p-values ≤ 0.001, ^**^ for p-values ≤ 0.01, ^*^ for p-values ≤ 0.05, ^•^ for p-values ≤ 0.10, and ^ns^ for p-values > 0.10

^†)^ All *β* is the long-run Okun coefficient arising as a sum of all short-run multipliers, regardless of their significance. Significant *β* is the sum of short-run multipliers significant at a 0.05 level of significance. ^‡)^ All regressors except the intercept and lag length are dummy variables. Dummy variables for countries, labelled as country effects, capture differences in Okun coefficients between countries. Bivariate filter, filter type and nonlinearity take a value of 1 (TRUE) when a bivariate adaptation of the filtering method is employed, when the UCM or H filter is used and when nonlinearity is detected by the Hansen test, respectively. Nonlinearity of Okun regressions is evaluated at a 0.05 significance level according to the tabular report in Appendix 3. The only numeric variable is lag length that varies between 0 and 4. In order to escape the trap of perfect collinearity, the country effect for Canada, the use of a univariate filter, the use of the HP filter and the absence of threshold nonlinearity pass together into the intercept. p-values are derived from standard errors corrected by the heteroskedasticity-consistent covariance matrix estimator of MacKinnon and White (1985) known as “HC3”

Regression of variability of Subperiod I–IV long-run multipliers $\beta$ upon configuration of filters and country factors

**Table:**

Response variable	Intercept	Country effects^‡)^						Bivariate filter^‡)^	Filter type^‡)^		R squared	# observations
		FR	DE	IT	JP	GB	US		UCM	H		
RMSE of all β^†)^	0.116^**^	0.099^**^	0.036^ ns^	0.044^ ns^	0.050^ ns^	0.043^ ns^	0.189^*^	0.043^ ns^	−0.081^ ns^	−0.067^•^	0.199	42
RMSE of significant β^†)^	0.166^*^	−0.021^ ns^	−0.026^ ns^	−0.033^ ns^	−0.007^ ns^	−0.037^ ns^	0.131^ ns^	0.058^ ns^	−0.035^ ns^	−0.033^ ns^	0.114	42

*Legend*: Significance labels displayed at computed statistics convey the following meaning: ^***^ for p-values ≤ 0.001, ^**^ for p-values ≤ 0.01, ^*^ for p-values ≤ 0.05, ^•^ for p-values ≤ 0.10, and ^ns^ for p-values > 0.10

^†)^ RMSE of all/significant $\beta$ used as the response variable is the root mean square error of all/significant Okun coefficients in Subperiods I, II, III and IV around the respective all/significant Okun coefficient for the whole period. Otherwise, all is the long-run Okun coefficient arising as a sum of all short-run multipliers, regardless of their significance. Significant $\beta$ is the sum of short-run multipliers significant at a 0.05 level of significance. ^‡)^ All regressors are dummy variables. Dummy variables for countries, labelled as country effects, capture different variability in Okun coefficients between countries. Bivariate filter and filter type take a value of 1 (TRUE) when a bivariate adaptation of the filtering method is employed and when the UCM or H filter is used, respectively. In order to escape the trap of perfect collinearity, the country effect for Canada, the use of a univariate filter, and the use of the HP filter pass together into the intercept. P-values are derived from standard errors corrected by the heteroskedasticity-consistent covariance matrix estimator of MacKinnon and White (1985) known as "HC3"

## Appendix 6: Comparison of Okun coefficients with other studies

Selected studies with one-regime linear regressions

**Table:**

Country	Moosa (1997) [UCM]			Lee (2000)			Ball et al. (2017) [HP]		Perman and Tavera (2005)	This study					
	OLS	Rolling OLS	SUR model	Kalman filter	HP filter	BN filter	Quarterly data	Annual data		HP filter		UCM filter		H filter	
										Univariate	Bivariate	Univariate	Bivariate	Univariate	Bivariate
CA	−0.601	−0.488	−0.492	−0.645	−0.633	−0.826	−0.524	−0.443		−0.504	−0.639	−0.108	−0.263	−0.381	−0.376
FR	−0.442	−0.217	−0.369	−0.400	−0.455	−0.344	−0.370	−0.353	−0.364	−0.090	−0.133	−0.024	−0.098	−0.177	−0.194
DE	−0.617	−0.410	−0.428	−0.581	−0.459	−0.565	−0.304	−0.363	−0.159	−0.149	−0.153	−0.100	−0.100	−0.222	−0.244
IT	−0.316	−0.175	−0.202	−3.846	−1.754	−0.415	−0.217	−0.295	−0.630	−0.088	−0.077	−0.132	−0.131	−0.160	−0.172
JP	−0.123	−0.083	−0.094	−0.079	−0.153	−0.182	−0.154	−0.165		−0.191	−0.190	−0.144	−0.144	−0.181	−0.210
GB	−0.479	−0.392	−0.389	−0.671	−0.709	−0.662	−0.360	−0.357	−0.681	−0.074	−0.076	−0.060	−0.004	−0.137	−0.146
US	−0.491	−0.456	−0.465	−0.532	−0.478	−0.493	−0.563	−0.476		−0.741	−0.877	−0.071	−0.713	−0.547	−0.568

*Note*: Okun coefficients reported in the table are assembled from Appendix 3 of this study and from four other studies. Moosa (1997) used annual data for 1960–1995 and estimated gap variables in a univariate UCM framework. His Okun coefficients are long-run multipliers from AR(1) OLS regressions (ibid., p. 349), averages from static OLS regressions applied on a rolling basis with a window of 14 years (ibid., p. 352), and coefficients from a SUR model (ibid., p. 353). Lee (2000, p. 341) made use of annual data for 1955–1996 (except Germany with 1960–1996) and estimated a statistic OLS regression. Variations consistent in different approaches to estimating gap variables: the Kalman filter augmented with a Phillips curve, the HP filter and the Beveridge–Nelson (BN) filter. Ball et al. (2017, p. 2431) applied to the HP filter to quarterly or annual data for 1980/Q1–2013/Q4 in order to estimate gaps. ARDL(0,2) models were applied to quarterly data and the reported Okun coefficients are long-run multipliers. Annual data were inserted into static regressions. Penman and Tavera (2005, p. 2506) based their analysis on biannual (semestrial) data for 1970/S1–2002/S2 in combination with the HP filter and ARDL modelling. Different lag lengths were applied to different countries, and coefficients were estimated in a SUR framework

*Caveat*: The comparability of Okun coefficients is hindered by different statistical frameworks and time spans adopted by the studies included in the table. Furthermore, inasmuch as Lee (2000) chose an opposite arrangement of the Okun equation (output gap as regressand, unemployment gap as regressor) his estimates were recomputed by taking a reciprocal transformation. In view of the caveat raised by Plosser and Schwert (1979), a reciprocal transformation neglects correlation between these variables, but suffices purposes of this comparison. This reciprocal transformation is also the method employed in the meta-analysis of research works on Okun’s law by Perman et al. (2015)

**Table:** Selected studies with two-regime linear regressions

Country	Silvapulle et al. (2004) [UCM]		Lee (2000)			Ball et al. (2017) [HP]	This study					
	OLS	M estimator	Kalman filter	HP filter	BN filter		HP filter		UCM filter		H filter	
							Bivariate	Univariate	Bivariate	Univariate	Bivariate	Univariate
*Up-regime*												
Canada			−0.571	−0.610	−0.847		−0.263	−0.491	−0.512	−0.261	−0.446	−0.115
France			−2.632	−0.503	−0.467		−0.339	−0.259	−0.495	−0.372	−0.156	−0.103
Germany			−0.909	−0.613	−0.840		−0.249	−0.049	−0.207	−0.364	−0.048	−0.206
Italy			−6.667	−1.961	−0.345		−0.302	−0.154	0.157	−0.285	−0.206	−0.084
Japan			−0.105	−0.157	−0.185		−0.108	−0.232	0.044	−0.066	−0.220	0.044
UK			−0.962	−0.775	−1.053		−0.212	−0.194	0.291	−0.213	−0.124	−0.080
USA	−0.318	−0.257	−0.575	−0.485	−0.518	−0.474	−0.244	−0.544	−0.629	−0.587	−0.623	0.131
*Down-regime*												
Canada			−0.943	−0.658	−0.578		−0.459	−0.637	−0.345	−0.474	−0.615	−0.082
France			−0.279	−0.410	−0.237		0.218	0.050	−0.048	0.185	0.031	0.013
Germany			−0.350	−0.366	−0.328		−0.008	−0.064	−0.124	−0.005	−0.056	−0.124
Italy			−1.493	−1.887	−0.439		0.127	0.086	−0.104	0.128	0.112	0.083
Japan			−0.022	−0.092	−0.217		−0.238	−0.276	−0.195	−0.154	−0.247	−0.195
UK			−0.641	−0.629	−0.649		0.019	0.037	−0.015	0.024	0.007	0.103
USA	−0.576	−0.614	−0.388	−0.467	−0.450	−0.587	−0.679	−0.726	−0.802	−0.755	−0.941	−0.101

*Note*: Okun coefficients reported in the table are assembled from Appendix 3 of this study and from three other studies. Silvapulle et al. (2004) estimated gap variables in an UCM mode with quarterly data for 1947/Q1–1999/Q4, and inserted gap estimates into a nonlinear ARDL(2,3) model with a zero threshold for the output gap. The reported Okun coefficients are long-run OLS and M (robust) estimates. Lee (2000, p. 341) made use of annual data for 1955–1996 (except Germany with 1960–1996) and estimated a statistic OLS regression. Variations consistent in different approaches to estimating gap variables: the Kalman filter augmented with a Phillips curve, the HP filter and the Beveridge–Nelson (BN) filter. Ball et al. (2017, p. 2431) applied to the HP filter to quarterly or annual data for 1980/Q1–2013/Q4 in order to estimate gaps. ARDL(0,2) models were applied to quarterly data and the reported Okun coefficients are long-run multipliers. Annual data were inserted into static regressions. Ball et al. (2017, p. 1422) used quarterly data for 1948/Q1–2013/Q4 in conjunction with the HP filter and ARDL(0,2) model. The reported Okun coefficients are long-run multipliers for non-recessions (up-regime) and recessions (down-regime)

*Caveat*: The comparability of Okun coefficients is hindered by different statistical frameworks and time spans adopted by the studies included in the table. Furthermore, inasmuch as Lee (2000) chose an opposite arrangement of the Okun equation (output gap as regressand, unemployment gap as regressor) his estimates were recomputed by taking a reciprocal transformation. In view of the caveat raised by Plosser and Schwert (1979), a reciprocal transformation neglects correlation between these variables, but suffices purposes of this comparison. This reciprocal transformation is also the method employed in the meta-analysis of research works on Okun’s law by Perman et al. (2015)

## Footnotes

1 Since traditionally *u* is measured in percentages and real output is expressed in logarithms of monetary units, the unemployment gap *u*^*c*^ measures a deviation from the unemployment rate in percentage points, and the output gap *y*^*c*^ is a continuously compounded percentage deviation from real output.

2 The volume of a matrix* W*_*n,k*_ answers to the geometrical volume of a parallelepiped $\cal{P}$ generated by edges stacked in* W*. Linear algebra (see Strang 2006, pp. 249–250) suggests that the volume of the parallelepiped $\cal{P}$ then satisfies the following relationship: *Vol*($\cal{P}$)^2^ = *det*(*WW*′). In consequence, the average volume of the parallelepiped must be understood relative to the number of edges, i.e. *AVol*($\cal{P}$): = *det*(*WW*′)^1/(2*n*)^. Here, of course, *AVol*(*W*) ≡ *AVol*($\cal{P}$).

3 The real GDP figures were downloaded from https://stats.oecd.org/Index.aspx?DataSetCode=QNA as LNBQRSA for Canada, France, Germany, the UK and the USA, and as VOBARSA for Italy and Japan (as of 25 Apr 2022). Reference years varied between 2012 and 2019. The unemployment rates were downloaded from https://data.oecd.org/unemp/unemployment-rate.htm (as of 25 Apr 2022).

4 This assertion is based on studying comparative box-plots of R squared values and on running an informal regression of R-squared values against the filtering method and time delay.

5 Focusing on the count of at least 3 insignificant subperiod Okun coefficients, it is the case of France (5 times for Subperiod I, 3 times for Subperiod II), Germany (5 times for Subperiod I), Italy (4 times for Subperiod II, 3 times for Subperiod III), Japan (4 times for Subperiod I), the United Kingdom (6 times for Subperiod II, 3 times for Subperiod III), and the United States (5 times for Subperiod I).

6 In particular, a subperiod R squared is smaller by more than 0.10 than the whole period R squared for Canada in Subperiods I and II (3 and 4 cases), for France in Subperiod I (1 case), for Germany in Subperiod I (3 cases), Italy in Subperiods I and IV (2 and 2 cases), Japan in Subperiod I and II (1 and 1 case), the UK in Subperiods I, II and IV (1, 3 and 1 cases), and the USA in Subperiods I, II and III (5, 3 and 1 cases).
